# Phytogalactolipid dLGG Inhibits Mouse Melanoma Brain Metastasis through Regulating Oxylipin Activity and Re-Programming Macrophage Polarity in the Tumor Microenvironment

**DOI:** 10.3390/cancers13164120

**Published:** 2021-08-16

**Authors:** Chung-Chih Yang, Meng-Ting Chang, Cheng-Kuei Chang, Lie-Fen Shyur

**Affiliations:** 1 Ph.D. Program for Cancer Molecular Biology and Drug Discovery, College of Medical Science and Technology, Taipei Medical University and Academia Sinica, Taipei 115, Taiwan; yangcc@narlabs.org.tw (C.-C.Y.); cck.ns@tmu.edu.tw (C.-K.C.); 2 Agricultural Biotechnology Research Center, Academia Sinica, Taipei 115, Taiwan; mengting@gate.sinica.edu.tw; 3 Department of Biochemical Science and Technology, National Taiwan University, Taipei 110, Taiwan; 4 Department of Neurosurgery, Shuang Ho Hospital, Taipei Medical University, New Taipei City 235, Taiwan; 5 Ph.D. Program in Translational Medicine, College of Medicine, Kaohsiung Medical University, Kaohsiung 807, Taiwan

**Keywords:** brain metastatic melanoma, galactolipid, oxylipin metabolome, macrophage polarization

## Abstract

**Simple Summary:**

Metastatic brain melanoma is a common metastatic cancer with a high mortality rate. Current clinical regimens use the anti-angiogenesis drug bevacizumab (Avastin) and/or Lipo-DOX, a drug capable penetrating the blood–brain barrier; however, both commonly result in adverse side effects and limited treatment results. This study provides evidence to support the function of a phyto-glyceroglycolipid, 1,2-di-*O*-α-linolenoyl-3-*O*-β-galactopyranosyl-*sn*-glycerol (dLGG) in inhibiting melanoma brain metastasis (MBM) in mice through reprogramming the tumor microenvironment and interacting with melanoma cells and macrophages. The novel function of oxylipin 9,10-EpOMEs + 12,13-EpOMEs in preventing melanoma cell invasion and microglia/macrophage distribution and polarization in the tumor microenvironment is presented. The novel anti-melanoma function and underlying molecular mechanism of dLGG proposed herein can be considered as a novel therapeutic strategy to combat MBM.

**Abstract:**

Current conventional cancer therapies for melanoma brain metastasis (MBM) remain ineffective. In this study, we demonstrated the bioefficacy of a phyto-glyceroglycolipid, 1,2-di-*O*-α-linolenoyl-3-*O*-β-galactopyranosyl-*sn*-glycerol (dLGG) alone, or in combination with liposomal doxorubicin (Lip-DOX) or Avastin against MBM in a syngeneic B16BM4*^COX−2/Luc^* brain-seeking melanoma mouse model. Treatment with dLGG–10, dLGG–25, dLGG–10 + Avastin–5, Lipo-DOX–2, dLGG–10 + Lipo-DOX–2 or Lipo-DOX–2 + Avastin–5 suppressed, respectively, 17.9%, 59.1%, 55.7%, 16.2%, 44.5% and 72.4% of MBM in mice relative to the untreated tumor control. Metastatic PD-L1+ melanoma cells, infiltration of M2-like macrophages and CD31+ endothelial cells, and high expression levels of 15-LOX/CYP450 4A enzymes in the brain tumor microenvironment of the tumor control mice were significantly attenuated in dLGG-treated mice; conversely, M1-like resident microglia and cytotoxic T cells were increased. A lipidomics study showed that dLGG promoted B16BM4 cells to secrete oxylipins 9,10-/12,13-EpOMEs into the culture medium. Furthermore, the conditioned medium of B16BM4 cells pretreated with dLGG or 9,10-EpOMEs + 12,13-EpOMEs drove M2-like macrophages to polarize into M1-like macrophages in vitro. An ex vivo 3D-culture assay further demonstrated that dLGG, 9,10-EpOME or 9,10-EpOME + 12,13-EpOME pretreatment attenuated B16BM4 cells invading brain tissue, and prevented microglia/macrophages infiltrating into the interface of melanoma plug and brain organ/tissue. In summary, this report provides a novel therapeutic strategy and mechanistic insights into phytogalactolipid dLGG for combating MBM.

## 1. Introduction

Epidemiological evidence shows that approximately 37% of cancer patients with metastatic melanoma develop melanoma brain metastasis (MBM), which causes high morbidity and mortality, with only about 4 to 5 months life expectancy after brain metastasis is diagnosed [1]. Different drug delivery strategies have been developed to cross the blood–brain barrier (BBB) and to increase drug availability and efficiency in the brain. For example, monotherapy using Lipo-DOX, stable vesicle-like liposome-encapsulated doxorubicin, an anti-cancer drug, was effective in treating metastatic melanoma in a phase II clinical trial [2,3]. Bevacizumab (Avastin), a monoclonal antibody directly targeting vascular endothelial growth factor-A (VEGF-A), has been widely used in clinical trials for patients with melanoma, HER2-negative breast cancer, and so on [4,5]. High levels of VEGFs, VEGF-A, VEGF-C and VEGF receptors (VEGFR-1, -2, and -3) are commonly identified in benign melanocytic tumors, malignant melanoma, and stromal cells surrounding tumors, e.g., macrophages, endothelial cells, and fibroblasts [6]. A combination of bevacizumab with an alkylating chemotherapeutic drug temozolomide as a first-line treatment had a suppressive effect on melanoma with uveal metastasis in a phase II study [7]. Although a combination of targeted therapy (e.g., BRAFi/MEKi) increased the intracranial response rate (58%) compared with the BRAF inhibitor only (25–40%) in MBM patients, the durability of the response rate was shorter than in patients with extracranial disease. Targeting the immune check point, PD-1 antibody therapy alone or combined with CTLA-4 could induce response rates of 20% and 55%, respectively, in MBM clinical treatment [8]. However, the effectiveness of target therapies remains unsatisfactory due to various issues, including melanoma stages, metastatic recurrence, drug resistance, and accompanying side effects [8,9].

In the central nervous system, microglia cells (F4/80+ or Iba1+) are important immune cells that serve as tissue-resident macrophages (TMEM119+) with M1-like (iNOS+) polarity that have a functional effect on brain development and neuron environment homeostasis [10,11,12]. On the other hand, the bone marrow-derived macrophages that infiltrate the brain tissue showing M2-like (CD163+) polarity might promote tumorigenesis by creating an immunosuppressive tumor microenvironment (TME) [10,11,12]. Tumor-associated macrophages (TAMs) are the most abundant leukocytes in tumors, and are attributed to cancer dissemination and metastasis, including breast cancer, gliomas, and melanoma [13]. Accumulating evidence has revealed that TAMs predominantly resemble M2-like polarized macrophages and interaction with melanoma cells and other stromal cells, such as neutrophils, Treg cells, and dendritic cells, dominate the immunosuppressive milieu in the TME [14]. The development of therapies that target TAMs to redirect the polarization from M2 to M1 phenotype in brain tumors, including glioma and melanoma, has been proposed [11,15]. Microglia/macrophages displaying M1 polarity have been shown to have a tumoricidal effect that provides a strategy for clinical cancer therapy [11,14,15,16].

Lipoxygenases (e.g., 5-LOX, 12/15-LOX), cytochrome P450s (e.g., CYP450 epoxygenases/hydrogenases), and cyclooxygenases (e.g., COX-2), which catalyze polyunsaturated fatty acids to form a group of bioactive lipid mediators named oxylipins, play a role in cancer progression and metastasis [17]. For instance, 12(S)-HETE and 15(S)-HETE derived from arachidonic acid (AA) by 12/15-LOX increase B16F10 melanoma cell adhesion and lung metastasis through activating the ERK and FAK signaling pathways [18]; CYP450-4A produced from TAMs with M2 phenotype promoted pre-metastatic niche formation and cancer metastasis in a 4T1 breast cancer and B16F10 melanoma spontaneous metastasis mouse model [19]. In a hepatocellular carcinoma lung metastatic mouse model, a higher level of 5-LOX and metabolite LTB4 were observed in infiltrated alveolar macrophages, which were recruited by the interstitial macrophage-derived CCL2 and further promoted tumor lung metastasis [20].

A glyceroglycolipid 1,2-di-*O*-α-linolenoyl-3-*O*-β-galactopyranosyl-*sn*-glycerol (dLGG) isolated from the medicinal plant *Crassocephalum rabens* (Asteraceae) has been demonstrated to have anti-inflammatory activity in sepsis mouse models and anti-cancer activity in an orthotopic melanoma mouse model [21,22]. Of note, dLGG suppressed B16 melanoma lung metastasis by maintaining pulmonary vasculature tight junction permeability that resulted in preventing melanoma cell extravasation into the lung in syngeneic mice [23]. In the present study, we demonstrate that dLGG effectively attenuates melanoma brain-seeking cell aggressive properties in vitro and in vivo by inhibiting M2-marophage assembly and melanoma cell infiltration and growth into brain parenchyma as seen in a 3D-organoid culture model. This study also provides evidence for the therapeutic effect of dLGG alone or in combination with current anti-cancer drugs Lip-DOX or Avastin against brain metastatic melanoma.

## 2. Materials and Methods

### 2.1. Cell Lines

The B16 murine melanoma cell line (NRAS mutation) and A375 human melanoma cell line (*BRAF*^V600E^ mutation) obtained from the American Type Culture Collection (ATCC, Manassas, VA, USA) were grown in the product sheet suggested medium with 10% heat-inactivated fetal bovine serum, 100 units/mL penicillin and 100 mg/mL streptomycin at 37 °C in a humidified 5% CO_2_ incubator. THP-1, a human monocytic cell line from ATCC, was grown in RPMI1640 (Thermo Fisher Scientific, Waltham, MA, USA) medium supplemented with 10% fetal bovine serum, 4.5 g/L glucose and 0.05 mM β-mercaptoethanol. Primary human umbilical vein endothelial cells (HUVECs) were a kind gift from Professor Li-Wha Wu from the Institute of Molecular Medicine, National Cheng Kung University, Taiwan [23]. HUVECs were cultured in EndoGRO-LS medium (EMD Millipore, Burlington, MA, USA). A stable B16*^COX−2/Luc^* cell clone established previously was used in the mouse melanoma brain metastasis experiments [21]. A stable A375 cell clone (designated A375*^eIF4g/Luc^*) carrying a *hEF1alpha-eIF4g* promoter-driven luciferase (pIF4g.As2.luc.bla) reporter gene was created by lentivirus transfection. After transfection of the reporter gene construct into A375 cells and a few rounds of antibiotic-based (Blasticidin S, 10 μg/mL; Invivogen, San Diego, CA, USA) selection, the A375*^eIF4g/Luc^* cell clone containing stably expressed luciferase gene/protein was obtained (approximately 1 × 10^6^ RLU/s/μg protein/1 × 10^5^ cells).

### 2.2. Isolation of dLGG

Whole *C. rabens* plants (voucher specimen CB001, Agricultural Biotechnology Research Center, Academia Sinica, Taipei, Taiwan) at the flower blooming stage were collected and the compound dLGG was isolated with >98% purity following previously described protocols [21].

### 2.3. Cell Viability Assay

Melanoma cells (5 × 10^3^ cells/well) were seeded in a 96-well plate for 12 h and incubated with the indicated concentration of compounds for 24 h. The 3-(4,5-Dimethylthiazol-2-yl)-2,5-diphenyl tetrazolium bromide (MTT)-based colorimetric assay was used to measure cell viability based on the quantification data of the MTT salt absorbance at 570 nm. Cell viability was calculated using the following formula: viable cell number (%) = OD_570_ (treated cell culture)/OD_570_ (vehicle control) × 100.

### 2.4. Cell Clonogenic Assay

Colony-forming cell growth was attained by growing the parental cells and brain-seeking cells on 24-well plates with the indicated treatments for 6 days [24]. The treated cell colonies were further stained with 0.1% (*w*/*v*) crystal violet and then dissolved in 20% (*v*/*v*) acetic acid solution for quantification by measuring absorbance at 595 nm. The percentage inhibition of compound treatment was compared with the vehicle control-treated cells.

### 2.5. Tube Formation Assay

Low-passage HUVECs (1 × 10^4^ cells/well) were grown in endothelial growth basal medium (EndoGRO-LS medium; Millipore, MA, USA) for 12 h in growth factor-reduced Matrigel (BD Biosciences, NJ, USA)-coated 96-well plates. The tube formation of HUVECs was stimulated by adding 100 ng/mL human recombinant VEGF (Millipore, Burlington, MA, USA) in the presence of vehicle or compound for 12 h. Phenotypic tube formation was monitored every hour from 0 h to 12 h by microscope (Zeiss Imager Z1).

### 2.6. Cell Cycle and Cell Apoptosis Assay

Brain-seeking melanoma cells (B16BM4*^COX−2/Luc^* and A375BM4*^eIF4g/Luc^*) were synchronized by incubation with culture medium with 1% FBS for 24 h, and further treated with 140 μM dLGG for 12 h and 24 h. The treated-cells were fixed with cold 70% ethanol and stained with propidium iodide (PI; Sigma-Aldrich, St. Louis, MO, USA) for 30 min at room temperature. The results were analyzed by flow cytometry (BD Accuri C6 flow cytometer). For apoptosis detection, the melanoma cells were seeded in a 10 cm culture dish and treated with 140 μM dLGG for 24 h and 48 h. The apoptotic fraction was stained with an FITC/Annexin V Apoptosis Detection Kit (BD Pharmingen, Franklin Lakes, NJ, USA) according to the manufacturer’s instructions and analyzed by flow cytometry.

### 2.7. Western Blotting

Total cellular proteins were prepared from cells as previously described [21]. Protein samples were resolved by 10% SDS-PAGE and then immunoblotting. Protein bands reacting to specific antibodies were visualized by enhanced chemiluminescence (Amersham ECL prime; RPN 2232, GE healthcare) with exposure to chemiluminescence light film (Amersham Hyperfilm ECL; 28906839, GE Healthcare). The expression level of reactive protein bands was further quantified using Image J and Image Studio Lite software (LI-COR Biosciences). Protein markers (TM-PM10170) and the anti-mouse IgG (H + L)-HRP (RA-BZ102) were purchased from Biotools (Biotools, Taipei, Taiwan). Horseradish peroxidase-conjugated secondary antibodies (IgG (H + L)-HRP; Cat. AP132P, AP106P, AP124P; Millipore) were used. The anti-rabbit IgG (H + L)-HRP was purchased from Merck Millipore (AP132P). Antibodies against PD-L1 (ab58810), TGF-β (ab66043), Ki-67 (ab15580), and Src (ab133283) were from Abcam. GABA_A_R-α3 (PA1-4714) was from Thermo. MEK1/2 (9146), phospho-MEK1/2 (2338), phospho-ERK1/2 (9106), and phospho-STAT3 (Tyr705) (9131) antibodies were from Cell Signaling Technology (Beverly, MA). Antibodies against ERK1 (sc-94), BCL-2 (sc-7382), c-Jun (sc-169), p-c-Jun (sc-16312), tyrosinase (7833), and STAT3 (sc-482) were from Santa Cruz Biotechnology (Santa Cruz, CA, USA). Anti-VEGF (19003-1-AP) antibody was from Proteintech Group (Proteintec, Rosemont, IL, USA) and phospho-SRC (GTX81151) antibody was from GeneTex (Irvine, CA, USA).

### 2.8. Differentiation of THP-1 into Macrophages

THP-1 cells (1 × 10^5^/well) were incubated with 162 nM 12-O-tetradecanoylphorbol-13-acetate (TPA; P8139, Sigma-Aldrich, St. Louis, MO, USA) for 24 h in 24 well plates. After activation by TPA, 50 ng/mL IFN-γ and 25 ng/mL IL-4 (11725-HNAS, 11846-HNAS; Sino Biological, PA, USA) were added and incubated for 48 h to promote THP-1 differentiation into M1 and M2 macrophages, respectively, as the positive control [25].

### 2.9. Animals

Male C57BL/6JNarl mice were bred and obtained from the National Laboratory Animal Center, Taiwan. Female NOD/SCID (NOD.CB17-Prkdcscid/IcrCrlBltw) mice were bred and obtained from the Laboratory Animal Core Facility, Agricultural Biotechnology Research Center (ABRC-LACF), Academia Sinica, Taiwan. All of the mice were given a standard laboratory diet and distilled H_2_O ad libitum and kept on a 12 h light/dark cycle at 22 ± 2 °C in ABRC-LACF [23]. All experimental protocols (Protocol # 13-05-552) are approved by the Institutional Animal Care and Utilization Committee (IACUC), Academia Sinica, Taiwan [23].

### 2.10. Establishment of the Melanoma Brain Metastatic Mouse Model and Brain-Seeking Melanoma Cell Lines

Experimental mice (5 weeks old) were anesthetized by intraperitoneal (i.p.) injection of zoletil (0.16 mg/kg) and restrained on the platform of a dissecting microscope. The mice received intracarotid (ica.) injection surgery through ligating the left carotid artery at the distal part near the heart by surgical sutures and the second ligature was tied into a slipknot proximal to the injection site. A PE-10 (Intramedictm, No. 427401) tube was inserted into the lumen of the blood vessel to connect with the artery to inject melanoma cells (1 × 10^5^ cells/100 μL PBS). Luciferase expression was measured every two days, starting from day 7 after tumor cell injection [23]. The male C57BL/6JNarl mice, in which positive signals were detected by IVIS system in the brain region after ica. injection of B16*^COX−2/Luc^* cells, were sacrificed at day 17. The brain tissues were taken out of the skull and cut into small pieces in a culture plate containing 5 mL Hank’s balanced salt solution (HBSS; Sigma-Aldrich, St. Louis, MO, USA) containing 5 mg/mL papaya enzyme (papain; Sigma-Aldrich, MO, USA), then incubated in a CO_2_ incubator for 15 min to release B16*^COX−2/Luc^* melanoma cells into the HBSS solution. Five milliliters of RPMI1640 medium were added to stop the enzyme reaction and the tissues/cells containing media were centrifuged at 100× *g* for 10 min. The tissue/cell pellets were re-suspended in fresh medium and then filtered through a 0.40 μm filter. The collected cell solution was cultured in a T150 culture flask with RPMI 1640 medium and replaced by fresh culture medium after 24 h. Acclimated brain-seeking melanoma cell lines were obtained from 4 cycles of ex vivo primary cell culture processes (Figure S1a), and the final brain metastatic cells (BM) for further study were designated as B16BM4*^COX−2/Luc^*. We further established human brain-seeking melanoma cells A375BM4*^eIF4g/Luc^* using NOD/SCID mice and the same procedure.

### 2.11. In Vivo Animal Study

The experimental schemes of the animal studies are shown in Figure S2a,b. Briefly, the 5-week-old C57BL/6JNarl male mice were divided into eight groups (*n* = 15 for each group): sham, tumor control, dLGG–10, dLGG–25, Lipo-DOX–2, Lipo-DOX–2+Avastin–5, dLGG–10 + Avastin–5 and dLGG–10 + Lipo-DOX–2. At day 0, the sham group mice received 0.1 mL PBS, and the other groups received B16BM4*^COX−2/Luc^* cells (1 × 10^5^ cells in 0.1 mL PBS) by ica. injection. Starting from day 7, the dLGG–10 and dLGG–25 group mice were given 10 and 25 mg/kg dLGG, respectively, by forced oral feeding (per os; p.o.) every day. The Lipo-DOX–2 group mice received 2 mg/kg Lipo-DOX by intraperitoneal injection (i.p.) every 7 days. The mice in the combination treatment groups received 2 mg/kg Lipo-DOX i.p. every 7 days plus 5 mg/kg Avastin i.p. every 4 days (Lipo-DOX–2 + Avastin–5), 10 mg/kg dLGG p.o. every day plus 5 mg/kg i.p. Avastin every 4 days (dLGG–10 + Avastin–5), or 10 mg/kg dLGG p.o. every day plus 2 mg/kg Lipo-DOX i.p. every 7 days (dLGG–10 + Lipo-DOX–2). The tumor group mice were given vehicle (0.2 mL PBS) by force feeding every day. The sham group mice received the same operation without any treatment.

The in vivo bioefficacy of dLGG against the human A375BM4*^eIF4g/Luc^* brain-seeking melanoma mouse model was studied by dividing NOD/SCID mice (5 weeks old) into three groups and *n* = 10 per group: sham, tumor control, and dLGG–25. At day 0, the sham group mice received 0.1 mL PBS and the other groups had A375BM4*^eIF4g/Luc^* cells (1 × 10^5^ cells/100 μL PBS) implanted by ica. injection. Starting from day 7, the tumor control group mice were given 0.2 mL PBS p.o. every day, and the dLGG–25 group mice received 25 mg/kg p.o. every day. The tumor control group mice were given vehicle (0.2 mL PBS) p.o. every day.

In vivo bioluminescence imaging of each tested mouse group was acquired using the IVIS spectrum system (Xenogen) every other day, starting from day 7 after tumor cell inoculation until the animals were sacrificed. Bioluminescent signals were quantified using Living Image 2.5 (Xenogen) as photons/sec/region of interest (ROI) [22].

### 2.12. Tight Junction Permeability Analysis

At 14 days after the tumor inoculation in the MBM model, experimental mice received FITC-conjugate dextran (fluorexcin isothiocyanate-dextran, Sigma) by tail injection and 30 min later, the mice were sacrificed by systemic perfusion. The brain tissues were collected and underwent 10% formalin-fixation and OCT-embedded preparation. After immunofluorescence staining, the CD31 protein and FITC-dextran were visualized by microscope (Zeiss Imager Z1) and quantified by AxioVision software (Carl Zeiss MicroImaging) [23].

### 2.13. Hematoxylin and Eosin Staining and Immunohistochemistry

The paraffin blocks of organ specimens from tested mice were prepared and sliced (4 μm), and subjected to hematoxylin and eosin (H&E) staining and 3,3′-diaminobenzidine (DAB, Novolink Polymer Detection Systems; Lieica Biosystems, Germany) staining based on previous procedures [23]. In addition, brain tissues were fixed in 10% formalin solution, dehydrated by 15% to 30% sucrose/PBS solution, and embedded into optimum cutting temperature compound (OCT; 3801480, Leica) for frozen tissue sections (6 μm). Organ tissue morphology and the measurement of the expression level of proteins of interest were conducted by immunohistochemistry (IHC) staining and visualized by fluorescent microscope (Zeiss Imager Z1) and AxioVision software (Carl Zeiss MicroImaging). Iba1 (10904-1-AP), CD163 (16646-1-AP), GFAP (16825-1-AP), and CD31 (11265-1-AP) antibodies were from the Proteintech Group (Proteintec, IL, USA). Neutrophil elastase (ab68672), FOX3/Neu N (ab104224), and Mel-A for melanoma (ab732) antibodies were from Abcam. F4/80 (123101) antibody was from BioLegend. iNOS/NOS type II (610328) antibody was from BD Transduction Laboratories. 15-Lipoxygenase (sc-133085) antibody was from Santa Cruz Biotechnology (Santa Cruz, CA, USA). The reacted target proteins were visualized by immunofluorescence dyes (goat anti-rabbit/mouse IgG H&L; Alexa Fluor 488, 594; ab150077, ab150116; Abcam).

### 2.14. Ex Vivo 3D Tumor-Brain Organoid Co-Culture Model

Brains were taken from 5-day-old mice and the brain coronal sections (1 mm) were prepared by rodent brain matrix (RBM-2000C; ASI instrument, Eugene, OR, USA). The brain slices were placed onto a 6-well plate cell culture insert (CAT.35306; SPL life Sciences, Korea) and incubated with 100 μL RPMI1640/DMEM culture medium overnight at 37 °C in a 5% CO_2_ incubator. Tumor plugs were prepared using B16BM4*^COX−2/Luc^* or A375BM4*^eIF4g/Luc^* cells (5 × 10^5^) with (pretreatment group) or without (post-treatment group) compound treatment for 24 h, mixed with ECM (Basement Membrane Extract; CAT.354234, BD Falcon); and filled into a sterilized metallic spacer (5 mm diameter) and incubated for 2 h at 37 °C in a 5% CO_2_ incubator. The tumor plug was placed in contact with the brain slice for 6 days and the compound-containing RPMI1640/DMEM culture medium was changed every 2 days [26]. Immunofluorescence staining with specific antibodies to targeting melanoma cells (Mel-A) and macrophages (F4/80+) was conducted and visualized by light and fluorescent microscopes (Zeiss Imager Z1) and quantified by AxioVision software (Carl Zeiss MicroImaging) [23].

### 2.15. Ultra-Performance Liquid Chromatography–Electrospray Ionization Tandem Mass Spectrometry Analysis of Serum Oxylipin Metabolome

Serum (70 μL) was collected immediately from sacrificed mice and added to extraction solvent containing CHCl3:MeOH (2:1), 0.1 M 2,6-di-tert-ubutyl-4-methylphenol (ACROS ORGANIC, MA), and 0.01 M triphenylphosphine (SIGMA). Internal oxylipin standards (0.1 ppm of DHA-d_5_, 9-HODE-d_4_, PGE2-d_4_, and 1 ppm of 14,15-EET-d_4_, EPA-d_5_, 20-HETE-d_6_, 5-HETE-d_8_) purchased from Cayman Chemical (Ann Arbor, MI, USA) were added into samples before extraction [23]. The organic layer was collected by centrifugation. Five biological serum samples/repeats and five technical repeats for each biological sample were analyzed using ultra-performance liquid chromatography–electrospray ionization tandem mass spectrometry (UPLC-ESI-MS/MS). The culture medium of B16BM4 cells was collected immediately after the indicated treatment times, and stored at −80 °C before sample preparation for mass spectrometry analysis. The collected cultural media were supplemented with 10 μL of internal standards (0.1 ppm of DHA-d_5_, 9-HODE-d_4_, and PGE2-d_4_; 1 ppm of 14,15-EET-d_11_, and 5-HETE-d_8_), and then extracted using Oasis HLB cartridge (solid-phase extraction). The column was conditioned first with 6 mL of 100% MeOH and then with 6 mL of ddH_2_O. After loading the sample, the column was washed with 10% MeOH (20 mL), and the oxylipins were then eluted with 6 mL of 100% MeOH. The eluent was dried out under vacuum and redissolved in 100% MeOH for UPLC-ESI-MS/MS analysis. Samples were separated with ACQUITY UPLC BEH C18 column (particle size 1.7 μm, 2.1 × 100 mm^2^, Waters; Milford, MA, USA) at a 400 μL/mL flow rate using a 25 min gradient for analysis; the mass spectrometry setting and operation were according to our previous study [23]. Accuracy and precision were analyzed with quality control (QC) samples for each experimental group. Chromatogram acquisition, detection of mass spectral peaks, and their waveform processing were performed using Thermo Xcalibur 2.1 SP1 software (Thermo Fisher Scientific, MA, USA). The peak area of each quantified ion was calculated and normalized to the peak area of the corresponding internal standards.

### 2.16. Statistical Analysis

Experimental data were expressed as mean ± SD by using PASW Statistics 18. The statistical significance of differences between treatments was determined by analysis of variance (ANOVA) with Fisher’s post hoc test and Kruskal–Wallis test. A *p* value of greater than 0.05 was considered to be statistically significant. Different letters represent significant differences (one-way ANOVA, *p* ≤ 0.05). Kaplan–Meier survival plots were compared using a log-rank test.

## 3. Results

### 3.1. Establishment of the Melanoma Brain Metastatic Mouse Model and Brain-Seeking Melanoma Cell

In order to assess the mechanism of melanoma cell brain metastasis and the deregulating activities of phytogalactolipid dLGG and Lipo-DOX, we established a B16 and B16*^COX−2/Luc^*-carrying luciferase reporter gene melanoma brain metastatic mouse model by ica. injection. Then, brain-seeking melanoma cells (designated BM) with specific and high brain metastasis frequency compared to the parental cells in the mouse brain were acclimated and obtained from four cycles of repeating primary melanoma cells culture (B16BM1–4 and B16BM1–4*^COX−2/Luc^*) (Figure S1a). In order to select and decide the most appropriate brain metastatic clone for in vivo animal study, we used Western blotting to examine the expression of proliferation and metastatic markers including p-Src, tyrosinase, TGF-β, GABA_A_R-α3, and Ki-67 in the B16, B16BM3 and B16BM4, and B16*^COX−2/Luc^*, B16BM3*^COX−2/Luc^* and B16BM4*^COX−2/Luc^* cell lines. Compared to parental cells, the BM cell lines (either B16BM3 and B16BM4 or B16BM3*^COX−2/Luc^* and B16BM4*^COX−2/Luc^*) had higher protein expression levels in most of the tested proliferation and metastasis-associated proteins (Figure S1b). We then established melanoma brain metastases in mice using B16BM4 and B16BM4*^COX−2/Luc^*. The bioimaging data in Figure S1c show that B16BM4*^COX−2/Luc^* cells were specifically detected in the mouse brain. The final survival time of mice carrying parental brain B16 melanoma was 17 days, and those of mice carrying B16BM4 and B16BM4*^COX−2/Luc^* were 13 days and 15 days, respectively (Figure S1d). We examined the coronal section of mouse brains and observed that most of the B16BM4/B16BM4*^COX−2/Luc^* tumors grew in mouse brain parenchyma, yet B16/B16*^COX−2/Luc^* tumors grew in the third ventricle and hippocampus area (Figure S1e). These data indicate that primary melanoma brain-seeking metastatic cell clones, B16BM4 and B16BM4*^COX−2/Luc^*, were successfully established. These clones were then used for subsequent studies in vitro and in vivo.

### 3.2. dLGG Inhibits B16BM4 Brain-Seeking Cell Activities, HUVEC Tube Formation, and Selected Marker Protein Expression

**Figure 1:**
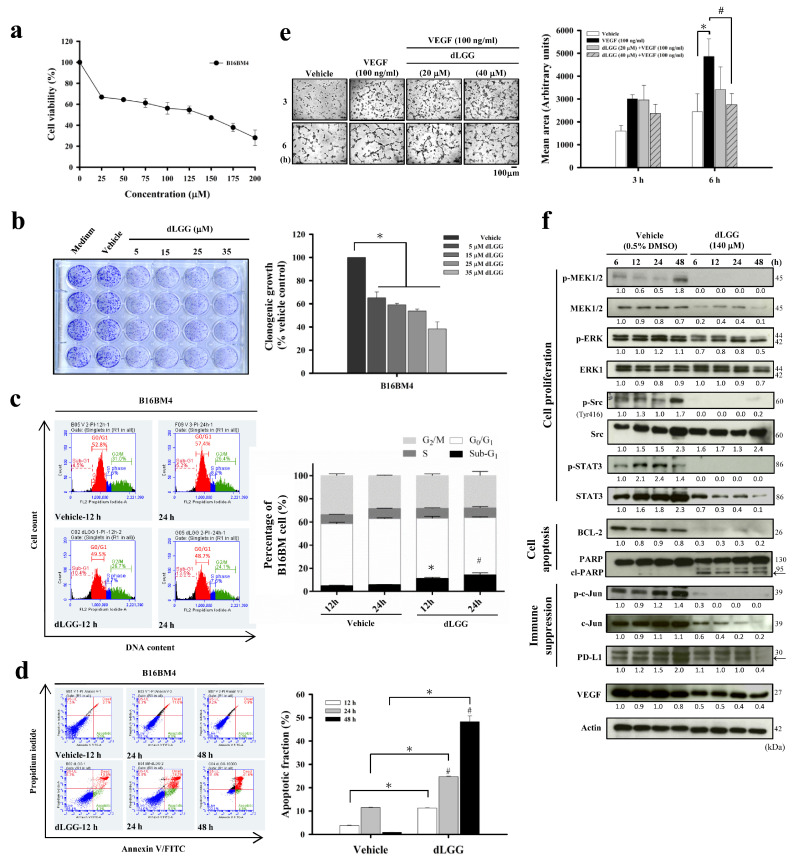
dLGG inhibits brain-seeking-cell proliferation and colony formation, and is anti-angiogenic. (**a**) The brain-seeking melanoma cells (B16BM4) were treated with vehicle (0.5% DMSO) or the indicated concentrations of dLGG at 37 °C for 24 h, and cell viability (%) was determined by MTT assay. (**b**) B16BM4 cells were treated with dLGG for 6 days. The colonies of melanoma cells were analyzed by crystal violet staining. Data are mean ± SD, *n* = 3; Kruskal–Wallis test; * *p* ≤ 0.05 compared with vehicle control; # *p* ≤ 0.05 compared with dLGG treatment for 12 h. (**c**) B16BM4 cells were treated with 140 μM dLGG for 12 h and 24 h before being stained with propidium iodide for cell cycle analysis using flow cytometry. Data are mean ± SD; *n* = 3; Kruskal–Wallis test; * *p* ≤ 0.05 compared with 12 h of vehicle control. # *p* ≤ 0.05 compared with 24 h of vehicle control. (**d**) B16BM4 cells were treated with 140 μM dLGG for 12, 24 and 48 h. Apoptotic cells were detected by annexin V/propidium iodide double staining and flow cytometry. Data are mean ± SD, *n* = 3; Kruskal–Wallis test; * *p* ≤ 0.05 compared with vehicle control. (**e**) The VEGF-stimulated (100 ng/mL) tube formation of primary HUVECs cells was observed after incubation for 6 h. The mean area of the vascular tube was analyzed by Image J software. Data are mean ± SD, *n* = 3; Kruskal–Wallis test; * *p* ≤ 0.05 compared with vehicle control. # *p* ≤ 0.05 compared with VEGF-treated group. (**f**) B16BM4 cells were treated with 140 μM dLGG for 6, 12, 24, and 48 h. The total proteins were prepared and subjected to Western blotting using the specific antibodies indicated. Actin protein intensity was used as the loading control. The target protein band intensity was analyzed by Image J software. Specific target protein expression level was normalized to respective actin band intensity first, followed by comparison with 6 h vehicle-treated protein band intensity.

We first investigated the in vitro bioactivity of dLGG against B16BM4 cells. The anti-B16BM4 cell (5000 cells/well) proliferation activity (IC_50_) of dLGG was determined to be 140.5 μM after treatment for 24 h (Figure 1a). The clonogenic ability of the B16BM4 cells was inhibited 35% to 62% when the lower melanoma cell population (500 cells/well) were treated with dLGG for 6 days at concentrations between 5 and 35 μM (Figure 1b). Further, when B16BM4 cells (8.6 × 10^5^ cells/10 cm culture dish) were treated with 140 μM dLGG for 24 h, the sub-G1-phase cell population was slightly induced (up to 11.8%) compared with the vehicle control (5.9%) as measured by a flow cytometer using a PI staining probe (Figure 1c). The G1 (49.2%) and G2/M (26.5%) phases in the dLGG treated-B16BM4 cells were similar to the vehicle-treated control cells (56.8% and 25.7%, respectively), and there was no difference in the S phase in any of the tested cells (Figure 1c). Furthermore, flow cytometry analysis using the annexin V and PI double staining method showed that dLGG treatment time-dependently induced apoptosis in B16BM4 cells. At 24 h and 48 h treatment, apoptotic cells were detected to be 24.8% and 48.3%, respectively, compared to vehicle control cells for which apoptotic cells made up 11.5% and 0.85%, respectively (Figure 1d).

Brain tumors are commonly observed to have high levels of angiogenesis and thus, anti-angiogenesis drugs are used as first-line therapy for patients with brain tumors [4,27,28]. We examined whether dLGG plays a role in inhibiting the VEGF-stimulated tube formation phenotype in primary HUVECs, thus representing the potential for anti-angiogenesis activity. When HUVECs were grown in Matrigel containing 100 ng/mL VEGF for 6 h, tube formation was significantly elevated (199%) compared to vehicle control cells (100%) (Figure 1e). When VEGF-stimulated cells were treated with 20 and 40 μM dLGG, the tube formation phenomena were inhibited 56.6–70% (Figure 1e).

We further examined the time-dependent effect of dLGG on the expression of some protein markers related to cell apoptosis, angiogenesis, and PD-L1 expression in B16BM4 cells. dLGG treatment significantly down-regulated anti-apoptotic Bcl-2 protein and increased the cleaved form of PARP, an apoptotic marker. dLGG also inhibited B16BM4 cells expressing VEGF (Figure 1f). Furthermore, dLGG significantly inhibited protein levels of PD-L1 and original forms or phosphorylated forms of MEK, ERK, c-Jun, Src and STAT3 in B16BM4 cells (Figure 1e). Together, our data demonstrated the in vitro effect of dLGG on anti-angiogenesis, and anti-cell proliferation and induction of apoptosis in melanoma brain-seeking cells.

### 3.3. dLGG Suppresses Melanoma Brain Metastases in Mice

**Figure 2:**
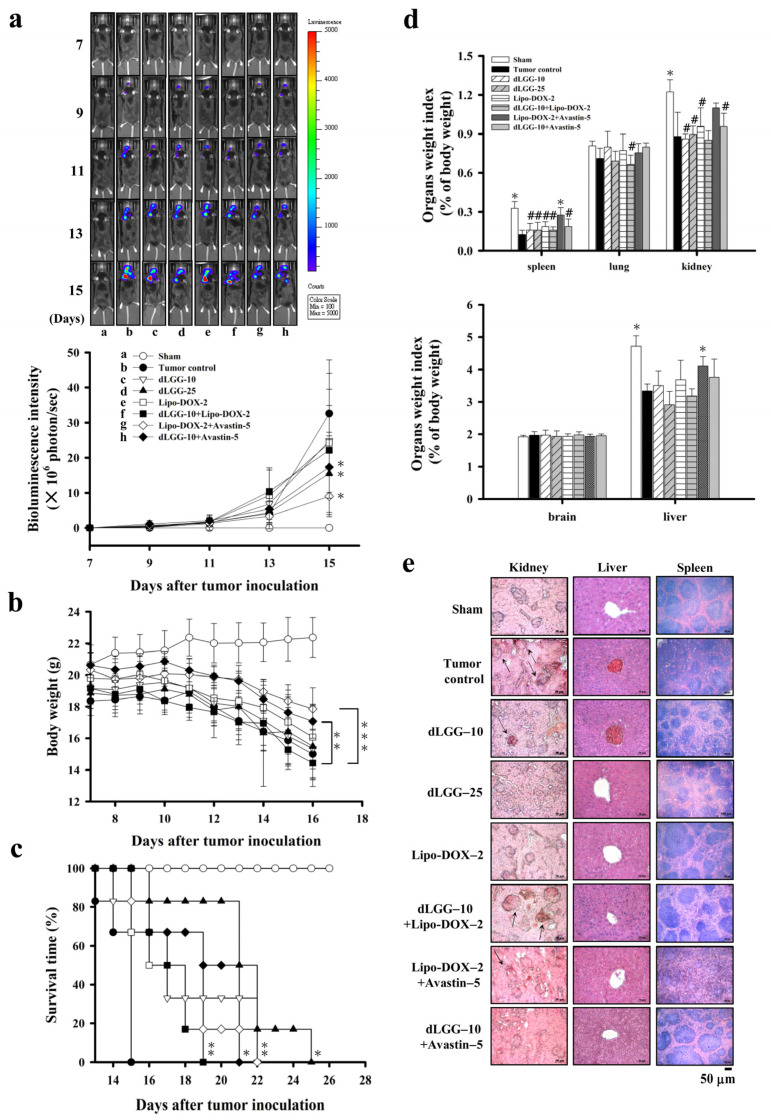
dLGG suppresses B16BM4^COX−2/Luc^ cell brain metastasis and prolongs mouse survival time. (**a**) Bioluminescence intensity of the 8 test groups of C57BL/6J mice with or without treatment were measured every other day starting from day 7 (two-way ANOVA, * *p* ≤ 0.05 compared with the tumor group). (**b**) The mouse body weight was measured every two days over the experimental period (two-way ANOVA; ** *p* ≤ 0.01, *** *p* ≤ 0.001). (**c**) The mouse survival time was analyzed by log-rank test (* *p* ≤ 0.05, ** *p* ≤ 0.01 compared with tumor group). (**d**) Organ weight index (%) = specific organ weight/body weight × 100. Data are mean ± SD, *n* = 5; Kruskal–Wallis test; * *p* ≤ 0.05, ** *p* ≤ 0.01 compared with tumor control. # *p* ≤ 0.05 compared with sham control. (**e**) Histopathological examination of the kidney, liver, and spleen organs of the 8 test groups of animals by H&E staining (*n* = 5).

Next, we explored the in vivo anti-brain melanoma effect of dLGG using brain-seeking B16BM4*^COX−2/Luc^* melanoma cells and the syngeneic brain metastasis mouse model established in-house. Lipo-DOX and Avastin were used as reference drugs and combined with dLGG for treatment of MBM. The experimental schemes of the brain metastatic melanoma model are shown in Figure S2a. The experimental animals were divided into eight groups: sham, tumor control, dLGG–10, dLGG–25, Lipo-DOX–2, Lipo-DOX–2 + Avastin–5, dLGG–10 + Avastin–5 and dLGG–10 + Lipo-DOX–2 after ica. injection of melanoma cells into mice. At treatment day 15, the dLGG–25 (40.9%), dLGG–10 + Avastin–5 (44.3%), Lipo-DOX–2 + Avastin–5 (27.6%), and dLGG–10 + Lipo-DOX–2 (55.5% with *p* ≤ 0.07) were observed to have much less metastatic brain B16BM4*^COX−2/Luc^* melanoma than the tumor control (100%) group (*p* ≤ 0.01) (Figure 2a), while the dLGG–10 (82.1%) and Lipo-DOX–2 (83.8%) treatments were less effective, as determined by quantified bioluminescence data. The tumor control and most treatment group mice showed a similar weight loss pattern over the experimental time period, but the Lipo-DOX–2 + Avastin–5 and dLGG–10 + Avastin–5 group mice had relatively less body weight loss than the tumor control group (Figure 2b). Meanwhile, the mean survival time of all compound- or drug-treated mice was prolonged with statistical significance relative to the tumor control mice (15 ± 1 days): dLGG–10 (18 ± 3 days, *p* ≤ 0.03), dLGG–25 (21 ± 3 days, *p* ≤ 0.0002), Lipo-DOX–2 (17 ± 2 days, *p* ≤ 0.013), dLGG–10 + Lipo-DOX–2 (18 ± 2 days, *p* ≤ 0.003), Lipo-DOX–2 + Avastin–5 (18 ± 2 days, *p* ≤ 0.004), and dLGG–10 + Avastin–5 (19 ± 2 days, *p* ≤ 0.001) (Figure 2c). We further examined the major organ weight index (organ weight/body weight) in all experimental mice. Sham control mice had a higher organ weight index for the spleen, kidney, and liver compared with the tumor control; and most of the treatment groups had lower indexes for the three major organs. The brain weight index showed no difference in any of the mouse groups and we did not observe peritumoral edema in the brains of any of the groups of mice (Figure 2d). We further assessed the tissue architecture of the spleen, kidney and liver for each group by H&E staining (Figure 2e). In the tumor control group mice, some destruction of the glomerular structure in the kidney, white pulp and red pulp structure in the spleen, and more infiltration of red blood cells in the kidney and liver central vein relative to sham control mice were observed. The dLGG–25 and dLGG–10 + Avastin–5 treatments in mice showed an organ protective effect compared to the tumor control with similar organ tissue structure to the sham group (Figure 2e). The Lipo-DOX–2 + Avastin–5 group of mice showed some apparent damage to the spleen and kidney tissues. Of note, the dLGG–10 + Lipo-DOX–2 treatment showed less spleen tissue damage compared with tumor control and Lipo-DOX–2 + Avastin–5 groups (Figure 2e).

### 3.4. dLGG Reprograms Tumor-Associated Macrophage Profiles in the Melanoma Tumor Microenvironment in Mouse Brain

**Figure 3:**
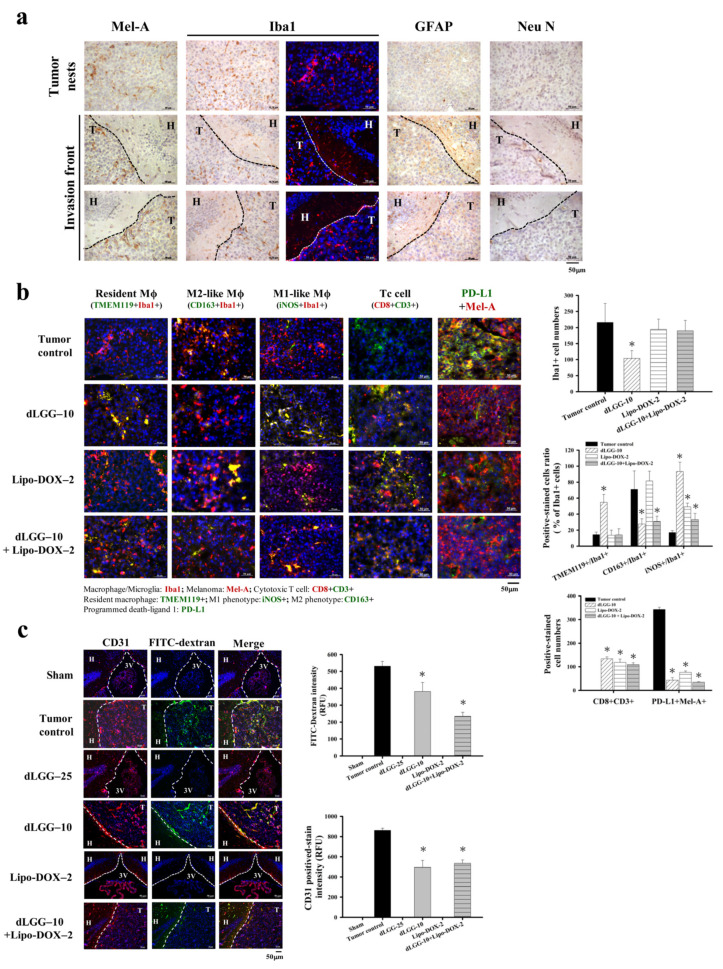
Various cell distributions and microglia/Mϕ polarity in the brain tissues of tested mice examined by immunohistochemistry. (**a**) Representative immunohistochemical images of mouse brain tissues with positive staining of melanoma cells (Mel-A), microglia/Mϕ (Iba1), astrocytes (GFAP), neurons (Neu N) (scale bar, 50 μm). T: tumor site, H: hippocampus. (**b**) The resident Mϕ (TMEM119+ Iba1+), M2-like Mϕ (CD163+ Iba1+), M1-like Mϕ (iNOS+ Iba1+), cytotoxic T cells (CD8+ CD3+), and the PD-L1 expression level of melanoma cells were assessed in brain melanoma tumor tissue of the tumor control, dLGG–10, Lipo-DOX–2-, and dLGG–10 + Lipo-DOX–2-treated mice were analyzed using immunofluorescence staining (scale bar, 50 μm). The positive-stained cells were quantified. Data are mean ± SD, *n* = 3; Kruskal–Wallis test; * *p* ≤ 0.05 compared with the tumor control. (**c**) The representative image of brain sections demonstrating the vasculature structure (CD31, red color) and the leakage of FITC-dextran (green color) in C57BL/6J mouse brains after 14 days of B16BM4*^COX−2/Luc^* cell inoculation. (scale bar, 50 μm). Data are mean ± SD, *n* = 3; Kruskal–Wallis test; * *p* ≤ 0.05 compared with the tumor control. T: tumor site, H: hippocampus, 3V: third ventricle.

Tumor-associated macrophages (TAMs) are the most abundant inflammatory cells, which orchestrate cancer development at different stages; thus, targeting TAMs has been considered to be a therapeutic strategy to combat various cancers [14]. We sought to determine whether the inhibitory mechanism of dLGG and Lipo-DOX against brain metastatic melanoma is through the re-education of TAMs and other immune cell types in the TME. First, we collected the coronal sections of brain specimens from all of the respective sham, tumor control and treated mice (Figure S3). We observed that in a few of the MBM mice, although the luminescent intensity was seen in the brain area of the mouse, few tumors actually grew at locations near the inner brain shell, or close to the nose, ear, and dura mater areas, except the brain parenchyma. To micro-dissect the immune cell type distribution in the brain microenvironment, we selected the mouse groups, including the tumor control, dLGG–10, Lipo-DOX–2, and dLGG–10 + Lipo-DOX–2 groups, which had at least three mice with a melanoma tumor growing in the brain parenchyma area (Figure S3) for IHC assays. We immune-stained the brain tissues of the tumor control mice for the population and expression of microglia/macrophage (Iba1), astrocyte (GFAP) and neuron (Neu N) cells along with melanoma cells (Mel-A). The Mel-A+ melanoma cells (DAB brown color) in the tumor nest and invasion front of mouse melanoma can be seen in Figure 3a. Notably, we observed that the activated microglia/macrophages (Iba1+) with round cell bodies were significantly accumulated in the tumor nest tissue and tumor invasion front site, as shown by brown DAB staining (left) or red immunofluorescent staining (right). Meanwhile, resting microglia/macrophages were also observed in the hippocampus area of brain tissue (Figure 3a). Most of the astrocytes (GFAP+) and neurons (Neu N+) were found localized in the hippocampus (H) of the brain tissue, but not found at the tumor site (T).

We further examined and compared the dynamics and polarity of microglia/macrophages (Mϕ) including resident Mϕ (TMEM119 + Iba1+), M2-like Mϕ (CD163 + Iba1+), and M1-like Mϕ (iNOS + Iba1+) in the TME of the tumor control and dLGG–10, Lipo-DOX–2 and dLGG–10 + Lipo-DOX–2 group mice. Of note, the overall Iba1+ cells in the brain tumor tissues of dLGG–10 mice were significantly attenuated by about 51.8% (104 ± 24 cells) compared with the tumor control (100%, 216 ± 59 cells), and there was little difference in Lipo-DOX–2 (90.1%, 194 ± 31 cells), and dLGG–10 + Lipo-DOX–2 (88.2%, 190 ± 32 cells) group mice (Figure 3b). Furthermore, 54.8% of the resident Mϕ (TMEM119 + Iba1+) cell population detected in the tumor tissues of dLGG–10 mice was demonstrated to have a higher distribution ratio than those in the tumor control (14.4%), Lipo-DOX–2 (13.6%), and dLGG–10 + Lipo-DOX–2 (14%) group mice (Figure 3b). Notably, only one mouse grew brain tumors in the dLGG–25 treatment group, in which a high expression level of resident Mϕ was observed compared to that of dLGG–10 and other treatment groups (data not shown). Meanwhile, we also observed that the TMEM119-non-resident macrophages were localized in the tumor tissues of each group of mice. The M2-like phenotype (CD163 + Iba1+) microglia/Mϕ in the tumor control (71%) and Lipo-DOX–2 (81.6%) mice displayed a higher distribution ratio than the dLGG–10 mice (27.7%) and dLGG–10 + Lipo-DOX–2 (31%) mice (Figure 3b). Moreover, 93.2% of the M1-like phenotype (iNOS + Iba1+) microglia/Mϕ distribution ratio in brain tumor tissues was significantly increased in the dLGG–10 mice compared to the tumor control (17%), Lipo-DOX–2 (49%), and dLGG–10 + Lipo-DOX–2 (33.4%) mice (Figure 3b). The results revealed that dLGG treatment alone or in combination with the anti-cancer drug Lipo-DOX–2 effectively facilitated the reprogramming of M2-like Mϕ into M1-like Mϕ population in the TME.

Furthermore, we also observed cytotoxic T cell (CD8 + CD3+) accumulation in the brain tumors of dLGG–10 mice (134 ± 8 cells), Lipo-DOX–2 (119 ± 14 cells), and dLGG–10 + Lipo-DOX–2 (109 ± 8 cells) compared to the tumor control group (not detected). In addition, the CD3+ positive-stained cells displayed a similar increased level in tumor tissues. Notably, PD-L1, an immune checkpoint protein expressed in melanoma cells (PD-L1 + Mel-A+) in brain tissues, was significantly decreased in dLGG–10 (44 ± 10 cells), Lipo-DOX–2 (76 ± 6 cells), and dLGG–10 + Lipo-DOX–2 (35 ± 2 cells)-treated mice compared with the tumor control (343 ± 9 cells) (Figure 3b).

### 3.5. dLGG Inhibits Angiogenesis and Prevents Melanoma Cell-Induced Vascular Tight Junction Permeability

We observed previously that B16 tumor cells enhance the permeability of vascular tight junctions in lungs, resulting in lung metastasis [23]. In this study, we assessed the BBB tight junction permeability of brain tumors in an MBM mouse model with or without dLGG/drug treatment. The FITC-dextran intensity in the melanoma brain tumor tissues represents the BBB and tight junction permeability in the MBM mouse model. The results show that dLGG–10 and dLGG–10 + Lip-DOX–2 treatments significantly decreased the FITC-dextran intensity in tumor tissues by 28.2% and 55.8%, respectively, compared to tumor control (Figure 3c). In addition, a high level of CD31+ vascular endothelial cells was observed in the tumor control (100%) that was significantly attenuated by dLGG–10 (42.6%) and dLGG–10 + Lip-DOX–2 (38%) treatments (Figure 3c).

### 3.6. Serum Oxylipin Metabolome in Tested Mice

**Figure 4:**
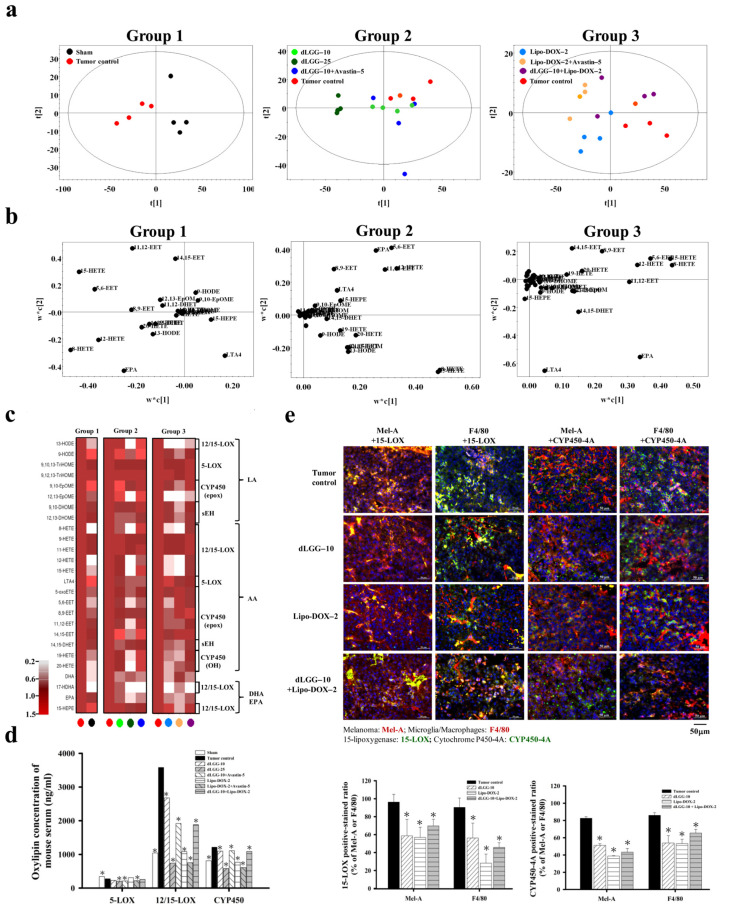
Oxylipin metabolite profile in melanoma brain metastasis mouse sera. (**a**) PLS-DA score plot and (**b**) loading plot of oxylipin profiles for Group 1: sham vs. tumor control; Group 2: tumor control vs. dLGG–10 vs. dLGG–25 vs. dLGG–10 + Avastin–5; Group 3: tumor control vs. Lipo-DOX–2 vs. Lipo-DOX–2 + Avastin–5 vs. dLGG–10 + Lipo-DOX–2. (**c**) Heat map showing the oxylipin metabolite intensity of sham and treated groups relative to tumor control arranged based on fatty acid substrates LA, AA, EPA, and DHA and the corresponding metabolic enzymes (*n* = 4 per treatment group and four technical repeats for each biological sample). (**d**) The summation of serum oxylipin concentration (ng/mL) derived from 5-LOX, 12/15-LOX, and CYP450 enzymes is shown. Data are mean ± SD, *n* = 4; Kruskal–Wallis test; * *p* ≤ 0.05 compared with tumor control. (**e**) Representative immunofluorescence images of mouse brain tissues with positive staining of 15-LOX and CYP450-4A in melanoma cell (Mel-A) and microglia/macrophages (F4/80). Data are mean ± SD, *n* = 3; Kruskal–Wallis test; * *p* ≤ 0.05 compared with the tumor control.

Oxylipins are an important group of lipid mediators that play a role in cancer metastasis [18]. We next examined whether the circulating oxylipins in BM mouse serum were regulated by the compound or drug treatment. Fifty-one oxylipin species were analyzed in the sera from the eight groups of mice using an oxylipin metabolomics platform established in-house and UPLC-MS/MS in a multiple reaction monitoring (MRM) model. Twenty-seven out of the 51 oxylipins detected with significant and reproducible levels in mice sera within the groups are summarized in Table 1 (by fold-change) and Table S1 (by quantified metabolite data). In order to gain insight into the oxylipin regulation in sham and tumor group mice and under dLGG or Lipo-DOX treatment (alone or in combination), we arranged the eight groups of mice into three groups for comparison: Group 1—sham and tumor control; Group 2—tumor control, dLGG–10, dLGG–25, and dLGG–10 + Avastin–5; and Group 3—tumor control, Lipo-DOX–2, Lipo-DOX–2 + Avastin–5 and dLGG–10 + Lipo-DOX–2. The quantified oxylipin data relative to retention times were analyzed by Thermo Xcalibur and SIMCA P+ software to generate a score plot and loading plot (Figure 4a,b) and a heat map was generated based on the fold-change of oxylipins in the three comparison groups (Figure 4c and Table 1). In Group 1, we observed that AA-derived 8-HETE, 12-HETE, and 15-HETE catalyzed by 12/15-LOX; AA-derived 5,6-EET, 11,12-EET, and 14,15-EET catalyzed by CYP450 epoxygenases; AA-derived LTA4 catalyzed by 5-LOX were all increased in the tumor control mice. The Group 2 data revealed that dLGG treatment deregulated oxylipins derived from AA in mouse sera, such as 8-HETE, 12-HETE, 15-HETE, 5,6-EET, 8,9-EET, and 11,12-EET and that those oxylipins were upregulated in the tumor group. In Group 3, Lipo-DOX–2 treatment also inhibited the HETEs (8-HETE and 12-HETE), EETs (5,6-EET, 8,9-EET, 11,12-EET, and 14,15-EET), and LTA4 levels compared with the tumor control (Figure 4b).

We further summed up the total oxylipin concentrations in the serum based on the respective catalytic enzymes, 5-LOX, 12/15-LOX, and CYP450 (epoxygenases and hydrogenases) (Figure 4d and Table S1). COX1/2-catalyzed oxylipins were detected at low levels and without significance within the groups, and are not shown or compared in this study. The levels of oxylipins detected in the tumor control group were designated as 100% (5-LOX derived: 275.4 ng/mL, 12/15-LOX derived: 3584.1 ng/mL, and CYP450 derived: 1213.1 ng/mL). The dLGG–10, dLGG–25, and dLGG–10 + Avastin–5 treatment inhibited 5-LOX-derived oxylipins to 83.8% (230.8 ng/mL), 74.2% (204.4 ng/mL), and 76.8% (211.4 ng/mL), respectively; 12/15-LOX-derived oxylipins to 74.8% (2681.3 ng/mL), 20.6% (737.6 ng/mL), and 53.3% (1916.2 ng/mL), respectively; CYP450-derived oxylipins to 90.6% (1099.3 ng/mL), 48.3% (585.8 ng/mL), and 91.4% (1108.3 ng/mL), respectively. The sera of the Lipo-DOX–2-, Lipo-DOX–2 + Avastin–5-, and dLGG–10 + Lipo-DOX–2-treated mice contained 5-LOX-derived oxylipins with slight or no inhibition; however, 20.2% inhibition in the Lipo-DOX–2 + Avastin–5 group and 5.7% in the dLGG–10 + Lipo-DOX–2 group were observed. Of note, 12/15-LOX-derived oxylipins were detected to be at a much lower level than the tumor control group, Lipo-DOX–2 group (30.2%), Lipo-DOX–2 + Avastin–5 (21%), and dLGG–10 + Lipo-DOX–2 group (52.4%). Meanwhile, CYP450 (epoxygenase/hydrogenase)-derived oxylipins were inhibited to 63.1% (765.5 ng/mL), 50.4% (611.5 ng/mL), and 89.7% (1087.3 ng/mL), respectively, relative to tumor control mice (100%, 1213.1 ng/mL). Taken together, we observed that the oxylipins derived from 12/15-LOX and CYP450 (epoxygenases/hydrogenases) that were significantly produced in the tumor control group were profoundly and dose-dependently inhibited by dLGG treatment (Figure 4d and Table S1).

### 3.7. dLGG Inhibits 15-LOX and CYP450-4A Expressions in Brain TME

Next, we examined the protein expression profile of 15-LOX and CYP450-4A (its metabolite 20-HETE was the most significantly inhibited by dLGG; 3-fold) in the brain TME of the tumor control, dLGG–10, Lipo-DOX–2 and dLGG–10 + Lipo-DOX–2 mice (*n* = 3 in each group) by IHC. The results in Figure 4e show that 15-LOX and CYP450-4A were produced by both microglia/Mϕ (F4/80+) and melanoma (Mel-A+) in brain tumor tissues; among them, the melanoma cells detected with 15-LOX+ (Mel-A+/15-LOX+) and Mϕ with 15-LOX+ (F4/80+/15-LOX+) were 364 ± 17 cells and 190 ± 6 cells, respectively, higher than CYP450-4A+ cell populations (Mel-A+/CYP450-4A+; 253 ± 15 cells, F4/80+/CYP450-4A+; 157 ± 6 cells). The three treatments that could inhibit melanoma or microglia/Mϕ cell populations also reduced expression of 15-LOX and CYP450-4A in the TME. dLGG–10, Lipo-DOX–2, and dLGG–10 + Lipo-DOX–2 inhibited the 15-LOX-positive-stained melanoma to 58.7%, 56.9%, and 69.6%, respectively, and 15-LOX-positive-microglia/Mϕ to 56.1%, 28.1%, and 45.6%, respectively, compared to the tumor control (Mel-A+/15-LOX+: 96.3%; F4/80+/15-LOX+: 90.3%) (Figure 4e). Meanwhile, dLGG–10, Lipo-DOX–2, and dLGG–10 + Lipo-DOX–2 inhibited the CYP450-4A-positive-stained melanoma to 51.1%, 38.7%, and 43.3%, respectively, and CYP450-4A-positive-microglia/Mϕ to 53.9%, 53.1%, and 65.5%, respectively, compared to the tumor control (Mel-A+/CYP450-4A+: 82.5%; F4/80+/CYP450-4A+: 86%) (Figure 4e). We also observed that in the dLGG–25 treatment group, 15-LOX and CYP450-4A in either melanoma cells or Mϕ were all significantly decreased (data not shown). These results suggest that enrichment of dLGG–10/–25 Lipo-DOX–2, or dLGG–10 + Lipo-DOX–2 significantly inhibited 15-LOX and CYP450-4A expression in Mϕ or melanoma.

### 3.8. dLGG-Primed Conditioning Medium of B16BM4 Cells Modulates Macrophage Polarity

**Figure 5:**
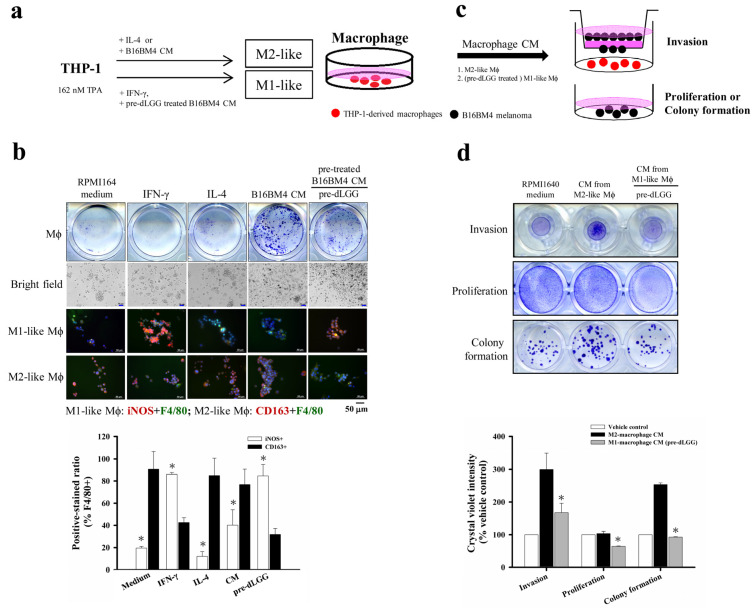
The bidirectional interaction of the Mϕ and the brain-seeking melanoma cells. (**a**) The schematic experimental design for THP-1 cells differentiated into Mϕ with either M1 or M2 phenotype with different cytokines or treatments. (**b**) The THP-1 cells differentiated into Mϕ by IFN-γ, IL-4, and the CM from melanoma pretreated with or without dLGG with 48 h incubation. The M1-like (iNOS + F4/80+) and M2-like (CD163 + F4/80+) phenotypic Mϕ were analyzed by immunofluorescence staining (scale bar, 50 μm). Data are mean ± SD, *n* = 3; Kruskal–Wallis test; * *p* ≤ 0.05 compared with CD163-positive-stained groups. (**c**,**d**) B16BM4 melanoma cells were cultured with the CM from M1/M2 Mϕ polarized by different treatments. The melanoma cell invasion ability was analyzed by transwell assay for 12 h. The B16BM4 cell proliferation and colony formation were analyzed by culturing with Mϕ CM for 24 h and 6 days. The B16BM4 cells were then fixed using 10% formaldehyde and stained with crystal violet. The quantification of crystal violet absorbance was detected at 595 nm. Data are mean ± SD, *n* = 3; Kruskal–Wallis test; * *p* ≤ 0.05 compared with vehicle control.

As the bidirectional interaction between microglia/Mϕ and melanoma cells is known to be involved in melanoma brain metastasis [29], we hypothesized that the culture medium from B16BM4 melanoma cell pretreatment with dLGG could affect Mϕ polarity and result in interference of the communication or interaction between Mϕ and melanoma cells, mimicking their relationship in the TME. We first prepared the M2-like phenotype of Mϕ control cells by using TPA (162 nM)-activated monocyte THP-1 cells, which were then differentiated into M2-like Mϕ (CD163+) cells by further incubating with IL-4 (25 ng/mL) for 48 h in RPMI1640 culture medium (Figure 5a); in parallel, another portion of the activated-THP-1 cells were differentiated into M1-like Mϕ (iNOS+) control by incubating with 50 ng/mL IFN-γ for 48 h in RPMI1640 culture medium (Figure 5a). Then, CM collected from B16BM4 cells, pretreated with vehicle or 140 μM dLGG for 24 h and then replenished with fresh medium for 12 h, was evaluated for its effect on Mϕ polarization using TPA-activated THP-1 cells. As shown in Figure 5b, after 48 h incubation with activated THP-1 cells, vehicle-treated B16BM4 CM converted more of the cells into M2-like Mϕ, and dLGG-pretreated B16BM4 CM significantly increased the ratio of M1-like Mϕ over M2-like Mϕ cell populations (Figure 5b).

We further evaluated the effect of THP-1-differentiated M2-like Mϕ (differentiated by incubating with B16BM4 CM) and THP-1-differentiated M1-like Mϕ (differentiated by incubating with CM from dLGG pretreated B16BM4) on B16BM4 cell proliferation, colony formation, and invasion ability. The two different phenotypes of THP-1-differentiated Mϕ cells were cultured in fresh RPMI1640 culture medium for 12 h and the CM were collected for subsequent assays (Figure 5c). The results in Figure 5d show that CM from M2-like Mϕ significantly promoted B16BM4 cell invasion (300%) and colony formation (253.2%) compared with RPMI1640 medium cultured cells (100%). Notably, the CM from M1-like Mϕ differentiated by the CM treatment collected from dLGG pretreated B16BM4 cells significantly inhibited B16BM4 cell invasion, proliferation, and colony formation to 55.8%, 62.5% and 36.6%, respectively, compared with the melanoma cell treated M2-like Mϕ CM (invasion: 100%, proliferation: 100%, and colony formation: 100%).

### 3.9. dLGG and Oxylipins 9,10-EpOME and 12,13-EpOME Inhibit Melanoma Cell Activity and Invade the Brain Parenchyma

We predicted that oxylipins present in the CM of melanoma cells might play a role in Mϕ polarization and melanoma–Mϕ interactions. We thus further analyzed the oxylipin profile/content in the CM from melanoma cells with/without dLGG pretreatment. On the basis of our UPLC/MS/MS data, dLGG (70 and 140 μM) treatment decreased the content of linoleic acid (LA) in the B16BM4 CM (Table S2). 13-HODE derived from LA catalyzed by 15-LOX, and 9,10-EpOME/12,13-EpOME derived from LA catalyzed by CYP450 (epoxygenase) in the B16BM4 CM were also significantly increased after pretreating with dLGG. In addition, the 9-HODE, 9,10,13-TriHOME, and 9,12,13-TriHOME derived from LA catalyzed by 9-LOX were also increased in the dLGG-pretreated B16BM4 CM compared to the vehicle control.

**Figure 6:**
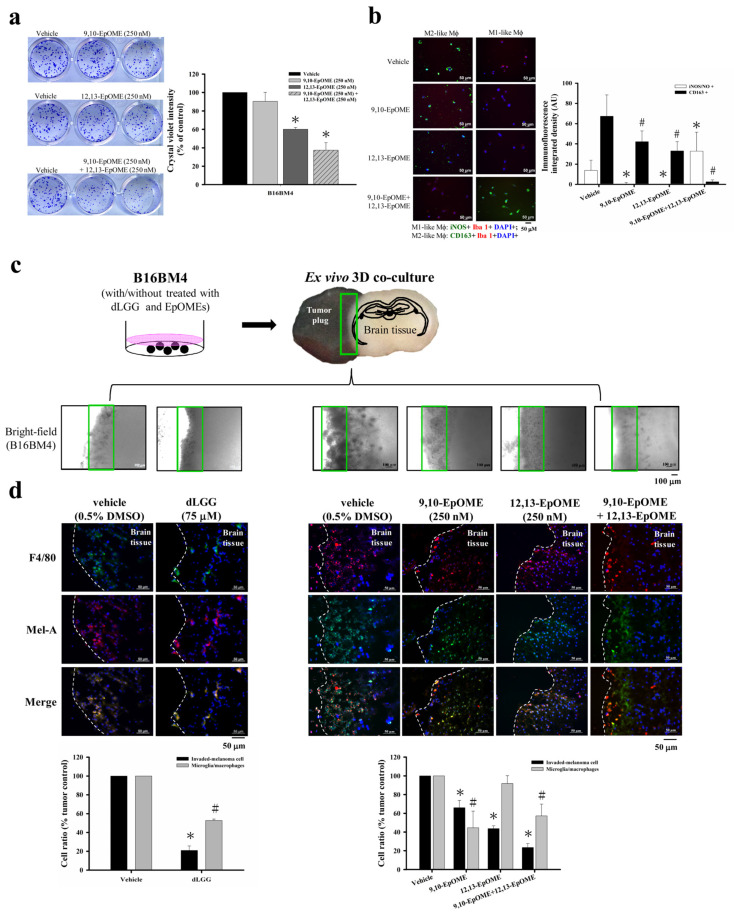
B16BM4 cells invading into brain tissue were suppressed by dLGG treatment. (**a**) 9,10-EpOME, 12,13-EpOME, and 9,10-EpOME + 12,13-EpOME inhibit B16BM4 cell colony-forming after incubation for 6-days. Data are mean ± SD, *n* = 3; Kruskal–Wallis test; * *p* ≤ 0.05 compared with vehicle group. (**b**) The THP-1 differentiated Mϕ displayed M1/M2 phenotype when incubated with medium containing EpOMEs for 48 h. M1-like Mϕ (green color: iNOS+; red color: Iba1+; blue color: DAPI+); M2-like Mϕ (green color: CD163+; red color: Iba1+; blue color: DAPI+). Data are mean ± SD, *n* = 3; Kruskal–Wallis test; * *p* ≤ 0.05 compared with the iNOS/NO+ group; # *p* ≤ 0.05 compared with CD163+ group. (**c**) A schematic diagram of ex vivo 3D-co-culture of the brain slice and the melanoma tumor plug. (**d**) B16BM4 cells with/without compound (75 μM dLGG) or specific oxylipin(s) (9,10-EpOME, 12,13-EpOME, 9,10-EpOME + 12,13-EpOME) treatment were used to prepare tumor plugs and then co-cultured with the brain tissue prepared from 5-day-old C57BL/6J mice for 6 days. The Mϕ (F4/80+) and melanoma (Mel-A) were detected by immunofluorescence staining. Data are mean ± SD, *n* = 5; Kruskal–Wallis test; * *p* ≤ 0.05 compared with the tumor control.

Because the function of 9,10-EpOME and 12,13-EpOME in melanoma progression remains unclear, we decided to examine whether 9,10-EpOME and 12,13-EpOME could directly inhibit melanoma progression, or whether they might play a role in macrophage polarization. A colony-forming assay was carried out to analyze the effect of 9,10-EpOME (250 nM) and 12,13-EpOME (250 nM) alone, or in combination (250 nM + 250 nM of each), on B16BM4 cells. The data in Figure 6a revealed that 6-day treatment with 12,13-EpOME (60.1%) or 9,10-EpOME + 12,13-EpOME (37.4%) significantly inhibited the B16BM4 cell colony-forming compared to vehicle (100%) or 9,10-EpOME (90.3%) treatment. Furthermore, we observed that the CM of 9,10-EpOME, 12,13-EpOME, and 9,10-EpOME + 12,13-EpOME pretreated B16BM4 cells suppressed the M2-like phenotype of Mϕ compared with the vehicle pretreated B16BM4 CM. Interestingly, the 9,10-EpOME + 12,13-EpOME combination-treated B16BM4 CM significantly promoted activated THP-1 cell differentiation into the M1-like phenotype after incubation for 24 h (Figure 6b). Our data imply that 9,10-EpOME and 12,13-EpOME might have an additive or synergistic effect.

A 3D-brain organ-tumor plug co-culture model was established to study whether dLGG or selected-oxylipins could affect melanoma crossing over the tumor–brain tissue barrier and preventing tumor growth in the brain parenchyma (Figure 6c). B16BM4 cells pretreated with 75 μM dLGG, 250 nM 9,10-EpOME, 250 nM 12,13-EpOME, and 250 nM 9,10-EpOME + 250 nM 12,13-EpOME, respectively, for 24 h were used to prepare tumor plugs. Then, the pretreatment cells were co-cultured with brain tissue slices in the medium containing vehicle (0.5% DMSO) or the same dose of dLGG, 9,10-EpOME, 12,13-EpOME, and 9,10-EpOME + 12,13-EpOME for 6 days. The culture media containing vehicle or dLGG/oxylipins were refreshed every 2 days. Light microscopy showed that dLGG, 9,10-EpOME, 12,13-EpOME, and 9,10-EpOME + 12,13-EpOME treatments inhibited B16BM4 cells invading brain tissues. The representative bright-field images are shown in Figure 6c. Furthermore, immunofluorescence analysis showed that only approximately 20.8% of dLGG-pretreated B16BM4 cells invaded into brain tissues compared to vehicle-treated cells (100%) (Figure 6d). B16BM4 cells with the following pretreatments 9,10-EpOME, 12,13-EpOME, and 9,10-EpOME + 12,13-EpOME invaded brain tissue at rates of 65.9%, 43.7%, and 23.4%, respectively. We also observed that in the 3D co-culture model, microglia/Mϕ (F4/80+) were activated (phenotypical swelling) and co-localized with invaded melanoma cells in the brain tissues. dLGG (52.8%), 9,10-EpOME (44.5%), and 9,10-EpOME + 12,13-EpOME (57.2%) treatments decreased the phenomenon of microglia/Mϕ cell infiltration compared with vehicle control (100%), while little effect was observed for 12,13-EpOME (91.8%) (Figure 6d). The function of both 9,10-EpOME and 12,13-EpOME in the inhibition of melanoma cell activity and invasion likely reflects the effect of dLGG in suppressing brain metastases in melanoma in mice.

## 4. Discussion

Melanoma metastases are the second most common type of brain metastases in humans. A high influx of tumor-associated macrophages (TAMs) in tumors is associated with poor prognosis for various cancers, including breast, ovarian, cervical, melanoma, and hepatocellular cancers [14]. On the other hand, massive accumulation of TAMs was able to consistently activate and facilitate the development of melanoma brain metastasis [13]. It is thus believed that brain metastases will be hampered by the elimination of accumulated TAMs at metastatic sites [30]. In the current study, we established brain-seeking B16BM4 melanoma cells and a syngeneic mouse model to demonstrate the bioefficacy of a bioactive phytogalactolipid dLGG. dLGG revealed moderate inhibition of infiltrated Mϕ (TEME119–Iba1+); however, dLGG reprogrammed the M2-like infiltrated Mϕ, seen abundantly in the TME of tumor control mice, into M1-like Mϕ. These data suggest that the M2-like Mϕ with pro-tumorigenic properties in melanoma brain tumor were attenuated by dLGG, supporting the notion of the inhibitory effect of dLGG on melanoma brain metastases. We used a 3D co-culture model to mimic the interactions of melanoma cells and microglial/Mϕ cells in vivo by observing the melanoma cells invading into the interface of the metastatic tissue and the brain parenchyma. We observed that dLGG pretreated melanoma cells inhibited Mϕ infiltration into the interface of the brain tissue contacting the tumor plug. These results demonstrated that dLGG prevented brain-seeking B16BM4 cells communicating and interacting with Mϕ in the brain tissue, further supporting the notion that dLGG inhibited melanoma brain metastases through reprogramming/re-educating Mϕ cells in the TME. We also established a xenograft human A375BM*^eIF4g/Luc^* melanoma in the NOD/SCID mouse model. The brain metastatic melanoma tumor growth was found to be significantly suppressed by dLGG treatment with the same treatment doses as those used in the syngeneic mouse B16BM4 melanoma in mice, and the treated mice had a longer survival time (data not shown). This study therefore demonstrates that dLGG did indeed have a therapeutic effect against mouse and human melanoma cells metastasized into animal brains.

Our results also demonstrate that a combination of a low dose of dLGG (10 mg/kg) with the anti-angiogenesis drug Avastin (5 mg/kg) (dLGG–10 + Avastin–5) also profoundly inhibited brain metastasis, and was more effective than treatment with a low dose of dLGG (10 mg/kg) alone. It has been reported that addition of bevacizumab to glioblastoma patients receiving radiotherapy and temozolomide treatment could extend progression-free survival but did not improve the overall survival time in phase III trials [31]. In this study, we observed that dLGG–10 + Avastin–5-treated animals had a longer survival time than the tumor control, suggesting the beneficial effect of the combination of Avastin with dLGG. Moreover, we also observed that dLGG–10 combined with Lipo-DOX–2 (dLGG–10 + Lipo-DOX–2) also showed greater inhibition of melanoma brain metastasis in animals when compared to animals treated with dLGG–10 or Lipo-DOX–2 alone. These data suggest that the natural phytogalactolipid, dLGG, in combination with the current anti-cancer drugs Lipo-DOX or Avastin, may be a useful strategy for combating melanoma brain metastasis in humans.

In this study, we investigated the systemic oxylipin metabolome in the sera of B16BM4-bearing mice and observed that dLGG significantly inhibited the levels of 12/15-LOX-derived oxylipins, such as 8-HETE, 12-HETE, and 15-HETE in the mice sera. A previous study reported that 12(S)-HETE promotes melanoma cell adhesion ability and metastasis potential by activating the ERK and FAK signaling pathways [18]. Meanwhile, 20-HETE derived from AA by CYP450-4A has been proposed to play a critical role in tumor growth and angiogenesis [32] that was detected less in dLGG–10/–25-treated (1–3-fold decrease) mouse serum than the tumor control mouse serum. The expression levels of both 15-LOX and CYP450-4A proteins from either melanoma (Mel-A+) or microglia/Mϕ (F4/80+) in the TME of dLGG–10- and dLGG–10 + Lipo-DOX–2-treated mice were lower than those in the tumor control mice. Our Western blotting data have also shown that dLGG treatment deceased the levels of 15-LOX and CYP450-4A in B16 melanoma cells (data not shown), supporting in part the decrease in both enzyme-derived oxylipins in dLGG-treated mice sera. Moreover, our study is the first to demonstrate that LA-derived oxylipins 9,10-EpOME and 12,13-EpOME have an inhibitory effect on melanoma cells invading the brain parenchyma. Moreover, 9,10-EpOME alone or in combination with 12,13-EpOME was able to promote M1-like Mϕ phenotype in vitro or in the brain melanoma tumor microenvironment. In addition to reprogramming the macrophage phenotype or activity, the observation of promotion of cytotoxic T cell infiltration and inhibition of angiogenic endothelial cell marker CD31 and VEGF protein expression in the TME sheds light on the novel modes of action of dLGG against melanoma brain metastases.

## 5. Conclusions

In summary, this study provides a novel therapeutic strategy to combat melanoma brain metastases by using a natural phytogalactolipid dLGG alone or in combination with the anti-cancer drug liposomal doxorubicin or the anti-angiogenesis drug Avastatin through targeting M1/M2 polarization and inhibiting angiogenesis in the mouse TME. The novel function of oxylipin 9,10-EpOMEs + 12,13-EpOMEs in preventing melanoma cell invasion and increasing M1-like phenotypic microglia/macrophages in the tumor microenvironment also reveals a potential mechanism for cancer therapy development.

## Figures and Tables

**Table 1:** Relative fold-change of oxylipins in the sera of melanoma brain metastatic mice with or without various treatments.

Substrates	Catalytic Enzyme	Oxylipins	Sham	Tumor	dLGG–10	dLGG–25	Lipo-DOX–2	Avastin–5 + Lipo-DOX–2	dLGG–10 + Lipo-DOX–2	dLGG–10 + Avastin–5
LA	15-LOX	13-HODE	0.5	1	1.0	0.3	0.3	0.2	0.5	1.2
	9-LOX	9-HODE	1.4	1	1.2	0.7	0.8	0.6	0.9	1.5
		9,10,13-TriHOME	0.9	1	0.9	0.9	0.9	0.9	1.0	0.9
		9,12,13-TriHOME	1.0	1	1.0	1.0	1.0	0.9	1.0	1.0
	CYP450 (epox)	9,10-EpOME	1.5	1	1.3	0.8	0.8	0.6	1.0	1.0
		12,13-EpOME	0.7	1	1.2	0.3	0.3	0.3	0.5	1.2
	sEH	9,10-DHOME	0.8	1	0.8	0.8	0.8	0.5	0.9	1.0
		12,13-DHOME	0.9	1	0.7	0.7	0.8	0.6	0.8	0.8
AA	12/15-LOX	8-HETE	0.3	1	0.7	0.2	0.4	0.3	0.8	1.1
		9-HETE	0.9	1	1.0	0.9	0.9	0.9	1.0	0.9
		11-HETE	0.9	1	1.0	0.9	0.9	0.9	1.0	1.0
		12-HETE	0.3	1	0.9	0.2	0.4	0.3	0.8	0.7
		15-HETE	0.5	1	0.9	0.2	0.4	0.3	0.8	1.1
	5-LOX	LTA_4_	1.3	1	0.8	0.7	1.1	0.8	0.9	0.7
		5-oxoETE	0.7	1	0.9	0.7	0.8	0.7	0.8	0.9
	CYP450 (epoxy)	5,6-EET	0.5	1	0.8	0.4	0.5	0.4	0.8	0.7
		8,9-EET	0.7	1	0.9	0.8	0.7	0.6	1.1	0.8
		11,12-EET	0.8	1	0.8	0.4	0.7	0.5	0.9	1.0
		14,15-EET	0.9	1	1.3	0.6	0.8	0.7	1.0	1.2
	sEH	11,12-DHET	0.8	1	0.8	0.4	0.7	0.5	0.9	1.0
		14,15-DHET	0.9	1	1.3	0.6	0.8	0.7	1.0	1.2
	CYP450 (OH)	19-HETE	0.6	1	0.9	0.4	0.6	0.5	0.9	1.1
		20-HETE	0.4	1	0.9	0.3	0.5	0.5	0.8	1.1
DHA			0.4	1	0.6	0.7	0.5	0.3	0.6	0.6
	12/15-LOX	17-HDHA	0.2	1	0.7	0.2	0.2	0.1	0.3	0.2
EPA			0.8	1	0.7	0.4	0.6	0.4	0.7	0.6
	12/15-LOX	15-HEPE	1.2	1	1.0	0.8	1.1	1.0	1.0	0.9

## Data Availability

The data presented in this study are available on request from the corresponding author.

## Supplementary Materials

The following are available online at https://www.mdpi.com/article/10.3390/cancers13164120/s1. Figure S1: Brain-seeking cells acclimated by repeating primary culture from an MBM mouse model with different batches of cell lines selected according to brain metastasis-related protein marker expression level by using Western blot analysis. Figure S2: The experimental scheme of the MBM mouse model. Figure S3: The coronal brain tissue sections in each experimental group were matched to the IVIS image of the corresponding mice in a B16BM4*^COX−2/Luc^* MBM mouse model. Table S1: Oxylipin metabolites identified in the sera of mouse-implanted brain metastatic melanoma after treatment with vehicle PBS, dLGG, Lipo-DOX, or combination treatments. Table S2: LA-derived oxylipins in the culture medium of B16BM4 cells with vehicle or dLGG treatments.
