# Supplementary material for: Phytogalactolipid dLGG Inhibits Mouse Melanoma Brain Metastasis through Regulating Oxylipin Activity and Re-Programming Macrophage Polarity in the Tumor Microenvironment
Source: Cancers (Basel). 2021 Aug 16;13(16):4120. doi: 10.3390/cancers13164120 (PMC8391228; doi:10.3390/cancers13164120)
Supplement: Supplementary file 1 [file cancers-13-04120-s001.zip › cancers-1313688-supplementary.pdf]

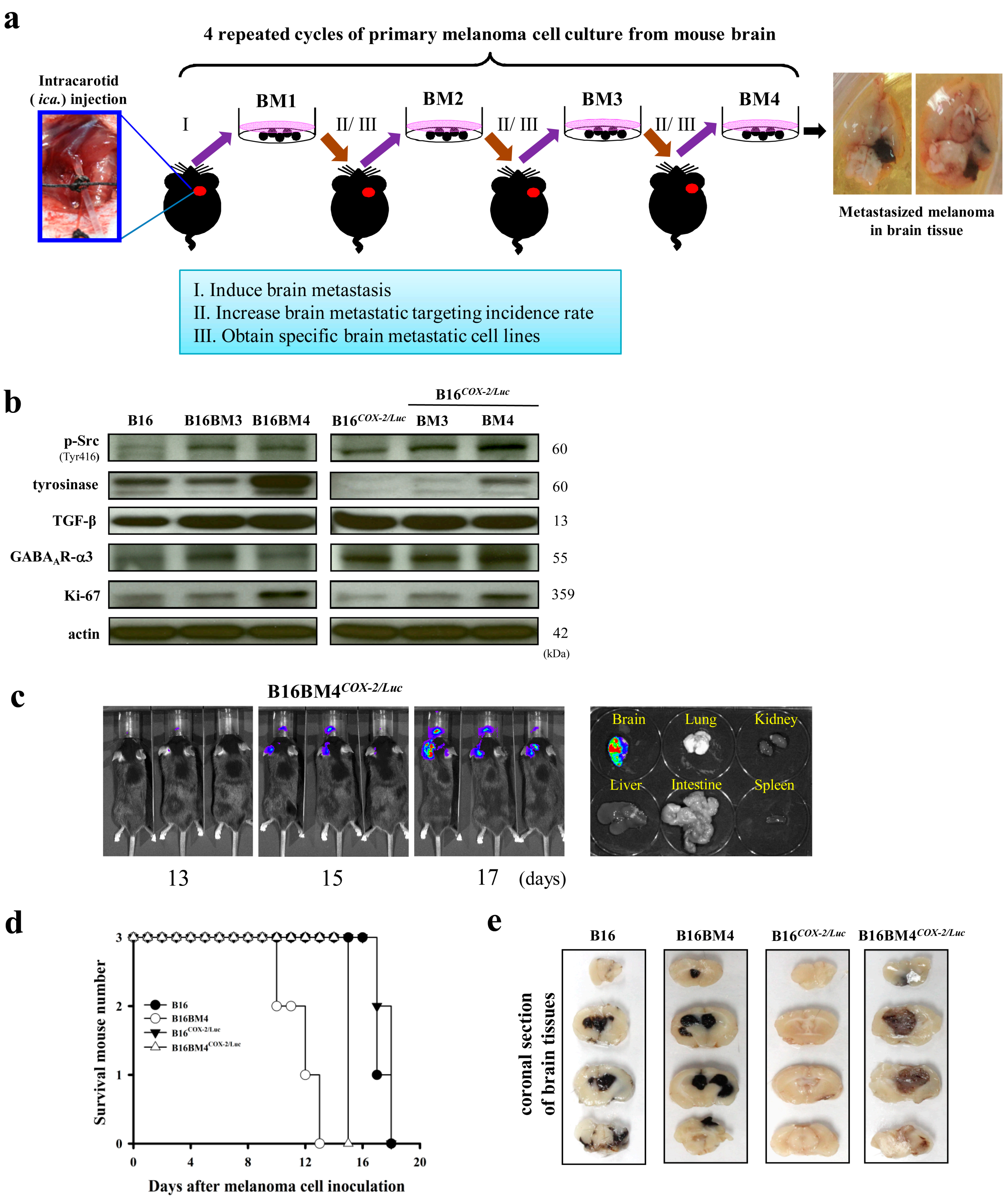
a
4 repeated cycles of primary melanoma cell culture from mouse brain
Intracarotid (ica.) injection
I
BM1
II/ III
BM2
II/ III
BM3
II/ III
BM4
Metastasized melanoma in brain tissue
I. Induce brain metastasis
II. Increase brain metastatic targeting incidence rate
III. Obtain specific brain metastatic cell lines
b
B16
B16BM3
B16BM4
B16COX-2/Luc
BM3
BM4
p-Src (Tyr416)
tyrosinase
TGF-β
GABAAR-α3
Ki-67
actin
60
60
13
55
359
42
(kDa)
c
B16BM4COX-2/Luc
13
15
17
(days)
Brain
Lung
Kidney
Liver
Intestine
Spleen
d
Survival mouse number
Days after melanoma cell inoculation
B16
B16BM4
B16COX-2/Luc
B16BM4COX-2/Luc
e
B16
B16BM4
B16COX-2/Luc
B16BM4COX-2/Luc
coronal section of brain tissues

Figure S1. Brain-seeking cells acclimated by repeating primary culture from MBM mouse model and the different batches cell lines were selected according to brain metastasis related protein markers expression level by using western blot analysis. (a) The experimental scheme for obtaining brain-seeking cells from MBM mouse model. (b) The brain metastasis related protein markers, such as p-Src, tyrosinase, TGF-β, $GABA_AR$-3α, and Ki-67, were used to select brain-seeking cells by western blot assay. (c) The MBM induced via ica. inject $B16^{COX-2/Luc}$. The brain metastasis in mouse was monitored by IVIS imaging system. The bioluminescence intensity of different organs were detected immediately when the mouse sacrificed. (d) MBM mouse survival rate. (e) The coronal brain tissue sections demonstrated melanoma brain metastasis and growth in the brain parenchyma.

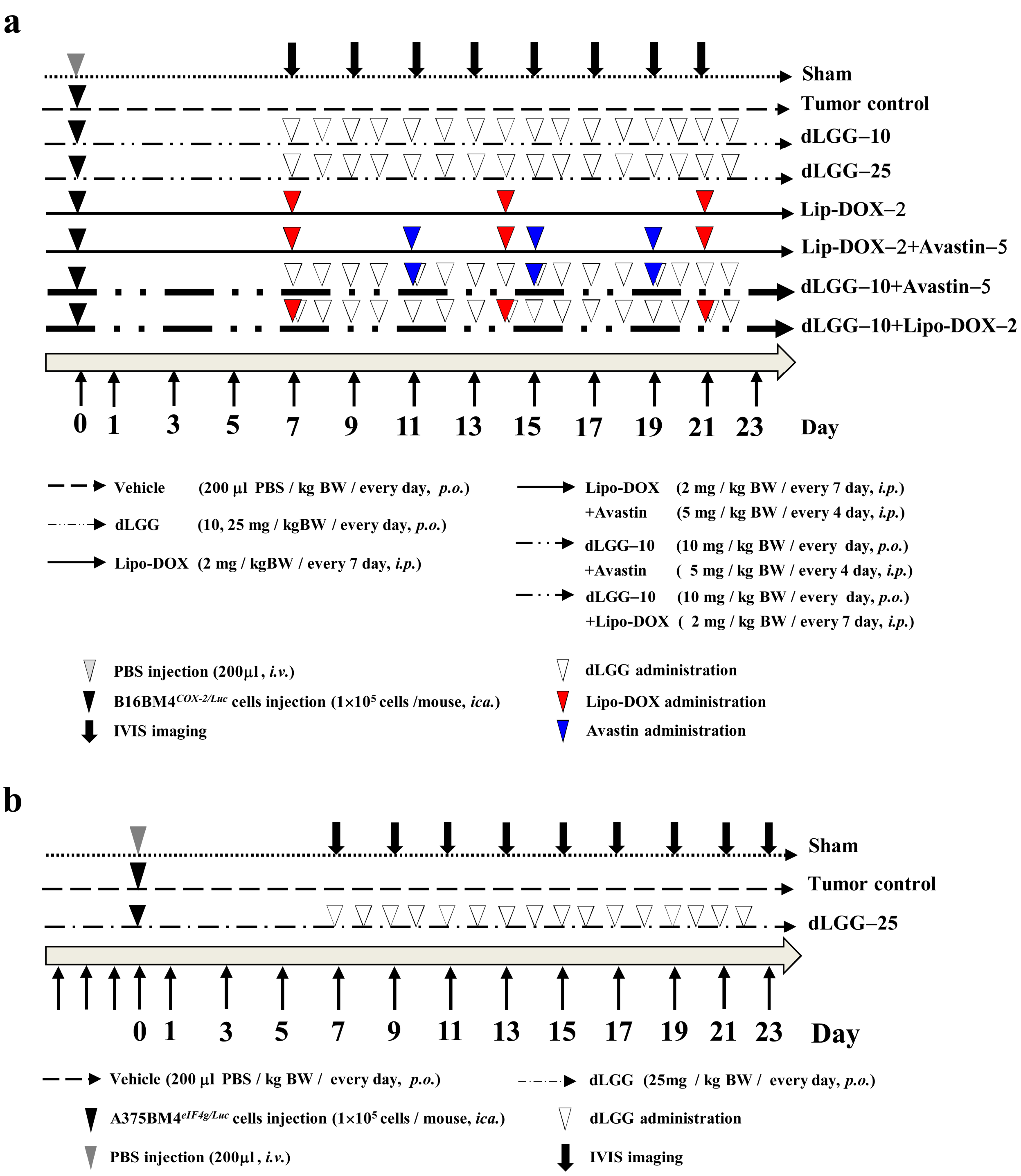
a
Sham
Tumor control
dLGG–10
dLGG–25
Lip-DOX–2
Lip-DOX–2+Avastin–5
dLGG–10+Avastin–5
dLGG–10+Lipo-DOX–2
0 1 3 5 7 9 11 13 15 17 19 21 23 Day
Vehicle (200 µl PBS / kg BW / every day, p.o.)
dLGG (10, 25 mg / kgBW / every day, p.o.)
Lipo-DOX (2 mg / kgBW / every 7 day, i.p.)
Lipo-DOX (2 mg / kg BW / every 7 day, i.p.)
+Avastin (5 mg / kg BW / every 4 day, i.p.)
dLGG–10 (10 mg / kg BW / every day, p.o.)
+Avastin (5 mg / kg BW / every 4 day, i.p.)
dLGG–10 (10 mg / kg BW / every day, p.o.)
+Lipo-DOX (2 mg / kg BW / every 7 day, i.p.)
PBS injection (200µl, i.v.)
B16BM4COX-2/Luc cells injection (1×10^5 cells /mouse, ica.)
IVIS imaging
dLGG administration
Lipo-DOX administration
Avastin administration
b
Sham
Tumor control
dLGG–25
0 1 3 5 7 9 11 13 15 17 19 21 23 Day
Vehicle (200 µl PBS / kg BW / every day, p.o.)
dLGG (25mg / kg BW / every day, p.o.)
A375BM4eIF4g/Luc cells injection (1×10^5 cells / mouse, ica.)
dLGG administration
PBS injection (200µl, i.v.)
IVIS imaging

Figure S2. The experimental scheme of MBM mouse model. (a) The MBM induced by *ica.* injecting B16BM4$^{COX\text{-}2/Luc}$ in C57BL/J6 mouse. (b) MBM induced by *ica.* injecting A375BM4$^{eIF4g/Luc}$ in NOD/SCID mouse.

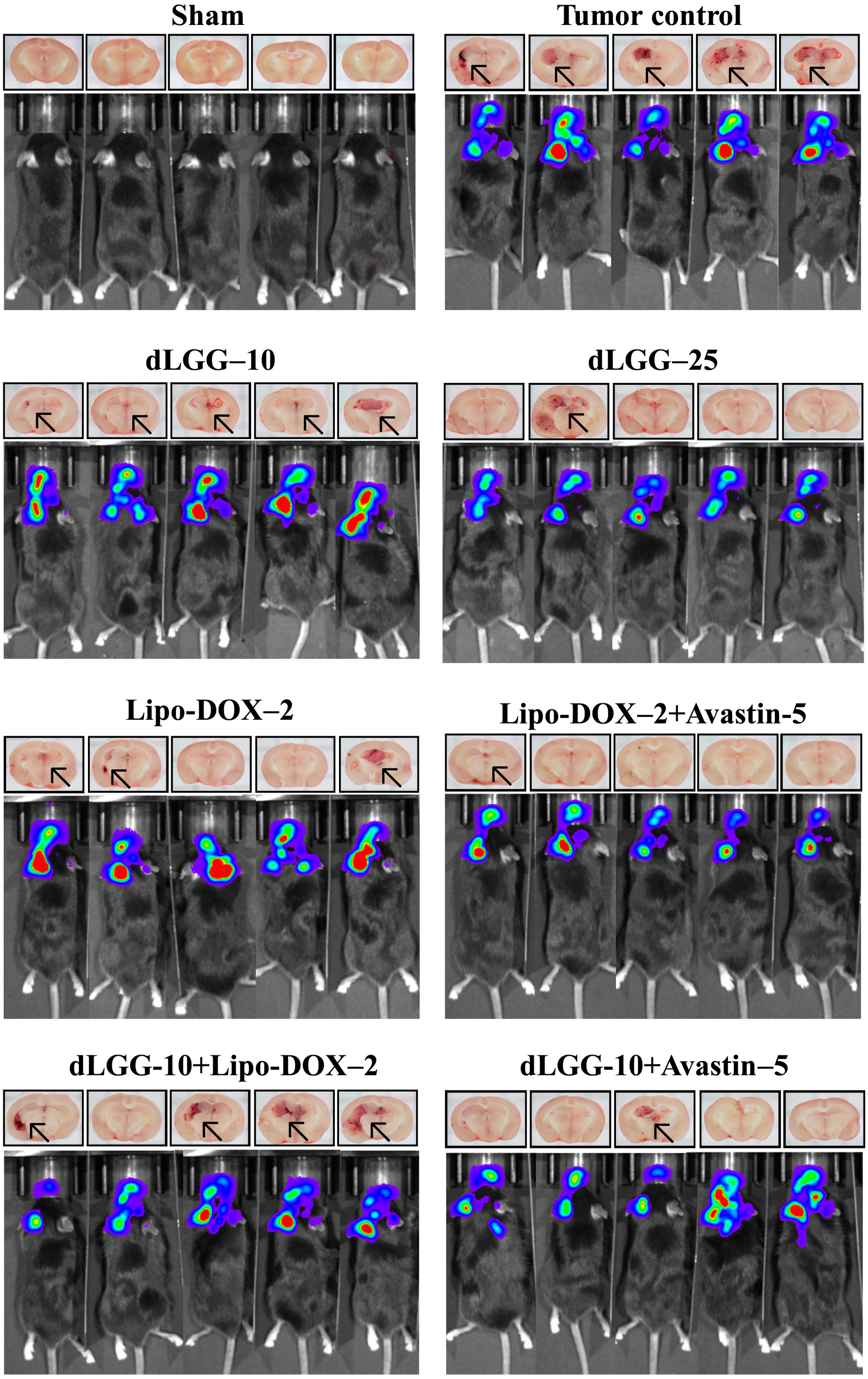

Sham
Tumor control
dLGG–10
dLGG–25
Lipo-DOX–2
Lipo-DOX–2+Avastin-5
dLGG-10+Lipo-DOX–2
dLGG-10+Avastin–5

Figure S3. The coronal brain tissue sections in each experimental group were matched to the IVIS image of the corresponding mice in a B16BM4$^{COX\text{-}2/Luc}$ MBM mouse model. The brain tissues were obtained from each groups of mice at the day 17 after brain B16BM4$^{COX\text{-}2/Luc}$ melanoma established. Each brain tissue was placed in the rodent brain matrix and sliced into coronal sections with identified position of brain area. The coronal section of brain specimens from all of the respective sham, tumor control, and dLGG, Lipo-DOX, dLGG+Avastin, dLGG+Lipo-DOX or Lipo-DOX+Avastin treated mice are shown.

Table S1

Oxylipin metabolites identified in the sera of mouse-implanted brain metastatic melanoma after treatment with vehicle PBS, dLGG, Lipo-Dox, or combination treatments.

	Sham	**Tumor control**	**dLGG–10**	**dLGG–25**	**Lipo-Dox–2**	**Lipo-Dox–2+ dLGG–10**	**Lipo-Dox–2+ Avastin–5**	**Avastin–5+ dLGG–10**
LA metabolites								
13-HODE	31.6 ± 9.3^{ab}	60.8 ± 12.7^{b}	58.7 ± 11.9^{b}	18.3 ± 3.68^{a}	18.0 ± 1.37^{a}	30.0 ± 14.4^{ab}	13.9 ± 1.08	70.9 ± 39.0ab
9-HODE	22.2 ± 5.52^{a}	16.0 ± 4.37^{abc}	19.2 ± 2.82^{ac}	10.6 ± 1.80^{b}	12.6 ± 1.26^{bc}	14.0 ± 3.33^{bc}	9.66 ± 0.96^{b}	24.0 ± 8.2^{ac}
9,10,13-TriHOME	5.5 ± 0.2^{a}	5.9 ± 0.1^{b}	5.5 ± 0.2^{a}	5.5 ± 0.2^{a}	5.4 ± 0.2^{a}	5.7 ± 0.2^{ab}	5.5 ± 0.2^{a}	5.3 ± 0.0^{a}
9,12,13-TriHOME	5.2 ± 0.1^{ab}	5.4 ± 0.1^{bc}	5.4 ± 0.3^{bc}	5.3 ± 0.1^{abc}	5.2 ± 0.04^{ab}	5.5 ± 0.3^{c}	5.2 ± 0.06^{a}	5.4 ± 0.1^{abc}
9,10-EpOME	26.5 ± 6.12^{a}	17.6 ± 3.72^{bc}	23.0 ± 3.31^{ab}	13.8 ± 2.83^{cd}	13.4 ± 1.22^{cd}	17.8 ± 4.00^{bc}	9.74 ± 0.28^{d}	17.8 ± 8.0^{cd}
12,13-EpOME	38.9 ± 10.5^{abc}	53.7 ± 12.8^{bc}	62.5 ± 10.7^{c}	17.0 ± 3.11^{a}	18.1 ± 1.60^{a}	28.5 ± 15.3^{ab}	13.6 ± 2.57^{a}	66.8 ± 31.1^{bc}
9,10-DHOME	12.00 ± 2.12^{ab}	14.68 ± 7.23^{b}	11.13 ± 1.76^{ab}	11.37 ± 5.23^{ab}	11.58 ± 0.51^{ab}	12.83 ± 1.58^{ab}	7.53 ± 0.58^{a}	14.8 ± 2.6^{ab}
12,13-DHOME	8.4 ± 1.6^{ab}	9.0 ± 2.4^{a}	6.7 ± 0.3^{bcd}	6.1 ± 0.6^{cd}	7.0 ± 0.3^{bcd}	7.5 ± 0.7^{ab}	5.5 ± 0.3^{d}	6.9 ± 0.2^{bcd}
AA metabolites								
8-HETE	147.0 ± 56.8^{a}	462.9 ± 88.8^{b}	332.0 ± 94.9^{bc}	83.6 ± 52.3^{a}	189.2 ± 70.5^{ac}	363.4 ± 128.2^{bc}	141.4 ± 48.5^{a}	494.9 ± 172.3^{b}
9-HETE	16.80 ± 0.18^{bc}	18.52 ± 0.55^{a}	17.61 ± 0.48^{c}	16.62 ± 0.13^{b}	17.23 ± 0.36^{bc}	17.79 ± 0.39^{c}	16.89 ± 0.36^{bc}	17.5 ± 0.5^{c}
11-HETE	17.5 ± 0.57^{abc}	18.5 ± 0.44^{c}	18.2 ± 0.46^{bc}	16.7 ± 0.16^{a}	17.4 ± 0.56^{ab}	18.3 ± 0.96^{bc}	17.2 ± 0.43^{ab}	18.0 ± 1.0^{abc}
12-HETE	92.3 ± 31.5^{a}	270.0 ± 52.0^{bd}	248.6 ± 66.7^{bd}	53.8 ± 28.2^{a}	117.1 ± 39.4^{a}	221.9 ± 74.9^{bcd}	90.0 ± 29.3^{a}	179.2 ± 93.5^{bc}
15-HETE	207.5 ± 98.8^{abc}	446.0 ± 99.1^{d}	412.5 ± 112.2^{d}	110.1 ± 28.1^{a}	191.4 ± 67.2^{abc}	375.7 ± 131.2^{bcd}	144.5 ± 47.1^{a}	489.8 ± 188.6^{d}
5-HETE	16.6 ± 0.03^{a}	16.7 ± 0.10^{bd}	16.6 ± 0.06^{abcd}	16.5 ± 0.01^{a}	16.6 ± 0.06^{abc}	16.6 ± 0.04^{bcd}	16.5 ± 0.02^{a}	16.7 ± 0.0^{cd}
LTA_4	311.2 ± 37.4^{a}	248.1 ± 10.6^{b}	200.7 ± 27.0^{cd}	183.0 ± 27.0^{d}	285.0 ± 19.6^{a}	234.3 ± 20.0^{bc}	199.3 ± 16.9^{cd}	176.6 ± 12.3^{d}
5-oxo-ETE	17.3 ± 0.24^{a}	23.8 ± 3.10^{c}	21.1 ± 0.62^{b}	17.3 ± 0.09^{a}	17.9 ± 0.40^{a}	20.1 ± 2.44^{ab}	17.5 ± 0.52^{a}	21.8 ± 1.3^{b}
5,6-EET	183.7 ± 49.7^{ac}	361.1 ± 79.8^{b}	286.0 ± 60.2^{bc}	131.3 ± 27.4^{a}	189.0 ± 73.2^{ac}	302.7 ± 121.1^{bc}	145.9 ± 40.1^{a}	254.2 ± 103.2^{ac}
8,9-EET	115.5 ± 12.4^{a}	176.0 ± 36.1^{a}	153.8 ± 30.8^{a}	135.6 ± 83.5^{a}	123.7 ± 29.4^{a}	188.0 ± 60.5^{a}	110.2 ± 14.2^{a}	141.6 ± 60.0^{a}
11,12-EET	247.2 ± 82.8^{abc}	319.7 ± 33.2^{c}	261.5 ± 45.5^{ac}	135.8 ± 32.6^{a}	222.9 ± 66.4^{abc}	279.7 ± 99.9^{bc}	160.0 ± 42.4^{ab}	311.7 ± 142.5^{abc}
14,15-EET	119.9 ± 22.1^{abc}	139.1 ± 26.7^{bcd}	176.2 ± 38.6^{d}	89.2 ± 5.09^{a}	112.0 ± 17.9^{abc}	145.3 ± 41.6^{cd}	95.4 ± 11.1^{ab}	165.3 ± 46.6^{cd}
14,15-DHET	117.1 ± 22.6^{ab}	150.7 ± 52.9^{b}	103.3 ± 5.59^{ab}	117.4 ± 52.6^{ab}	109.9 ± 8.84^{ab}	116.6 ± 22.0^{ab}	89.0 ± 4.8^{a}	142.4 ± 4.2^{ab}
19-HETE	25.3 ± 3.7^{a}	45.8 ± 6.5^{b}	43.2 ± 7.77^{b}	20.5 ± 3.06^{a}	28.5 ± 5.50^{a}	41.2 ± 8.20^{b}	24.4 ± 3.12^{a}	48.3 ± 12.5^{b}
20-HETE	34.0 ± 7.21^{a}	76.4 ± 15.6^{c}	72.1 ± 15.0^{c}	25.2 ± 6.7^{5a}	40.1 ± 9.87^{ab}	64.5 ± 17.2^{bc}	34.8 ± 8.2^{a}	80.7 ± 24.5^{c}
DHA metabolites								
DHA	6726.5 ± 651.4^{ac}	15465.5 ± 4095.9^{b}	9011.6 ± 684.0^{ac}	10252.0 ± 4005.0^{c}	8117.7 ± 845.5^{ac}	9487.0 ± 3514.5^{c}	4843.2 ± 897.8^{a}	9029.7 ± 3205.5^{ac}
17-HDHA	364.1 ± 215.1^{a}	2182.1 ± 268.82^{b}	1464.5 ± 305.1^{c}	347.6 ± 98.2^{a}	386.1 ± 192.1^{a}	718.3 ± 637.8^{a}	198.7 ± 102.9^{a}	538.9 ± 377.7^{a}
EPA metabolites								
EPA	332.2 ± 54.9^{a}	428.3 ± 99.6^{b}	279.4 ± 40.5^{ac}	183.6 ± 22.1^{acd}	244.8 ± 13.7^{acd}	281.8 ± 106.2^{ac}	171.2 ± 32.3^{d}	242.5 ± 57.5^{acd}
15-HEPE	198.0 ± 20.7^{a}	162.4 ± 6.6^{b}	165.0 ± 14.7^{b}	124.2 ± 6.78^{c}	179.7 ± 10.6^{b}	170.4 ± 4.2^{b}	163.1 ± 4.0^{b}	142.5 ± 12.3^{d}

Mouse blood was collected from heart, and serum flash-frozen in -80°C immediately after centrifuge isolation. Groups are defined as Sham (normal mouse), Tumor control (vehicle control: PBS with 5% DMSO), 10 mg/kg dLGG (dLGG-10), 20 mg/kg dLGG (dLGG-20), 2 mg/kg Liposomal doxorubicin (Lipo-Dox–2), and 5 mg/kg Avastin (Avastin–5). Data are means ± SEM (ng/ml; $n = 4$, $P < 0.05$, ANOVA, *post hoc* LSD).

Table S2

LA-derived oxylipins in the culture medium of B16BM4 cells with vehicle or dLGG treatments.

Substrate	Catalytic enzyme	Oxylipins	Vehicle	dLGG –70	dLGG –140
LA			62496.3 ± 503.2[a]	25682.9 ± 252.4[a]	55178.5 ± 395.0[a]
	15-LOX	13-HODE	0[a]	1880.7 ± 150.0[b]	4413.5 ± 256.6[c]
	9-LOX	9-HODE	0[a]	3873.1 ± 208.4[b]	5952.5 ± 1283.3[b]
		9,10,13-TriHOME	0[a]	1650.3 ± 181.3[b]	1890.8 ± 986.7[b]
		9,12,13-TriHOME	0[a]	1350.9 ± 169.3[b]	1872.1 ± 526.3[b]
	CYP450 (epoxygenase)	9,10-EpOME	0[a]	3785.6 ± 208.0[b]	5812.4 ± 1285.1[c]
		12,13-EpOME	0[a]	1833.8 ± 153.3[b]	4230.2 ± 247.1[c]

The culture media were immediately collected after 24 h treatment with vehicle, 70 µM dLGG (dLGG–70), or 140 µM dLGG (dLGG–140), and then subjected to solid-phase column extraction and LC/MS/MS analysis. Data are mean ± SEM (pg/ml; *n* = 3).
